# ASTRAL-III: polynomial time species tree reconstruction from partially resolved gene trees

**DOI:** 10.1186/s12859-018-2129-y

**Published:** 2018-05-08

**Authors:** Chao Zhang, Maryam Rabiee, Erfan Sayyari, Siavash Mirarab

**Affiliations:** 1 Department of Electrical and Computer Engineering, University of California at San Diego, 9500 Gilman Drive, La Jolla, 92093-0021 CA USA; 2 Department of Computer Science and Engineering, University of California at San Diego, 9500 Gilman Drive, La Jolla, 92093-0021 CA USA; 3 Bioinformatics and Systems Biology, University of California at San Diego, 9500 Gilman Drive, La Jolla, 92093-0021 CA USA

**Keywords:** Phylogenomics, Incomplete lineage sorting, ASTRAL

## Abstract

**Background:**

Evolutionary histories can be discordant across the genome, and such discordances need to be considered in reconstructing the species phylogeny. ASTRAL is one of the leading methods for inferring species trees from gene trees while accounting for gene tree discordance. ASTRAL uses dynamic programming to search for the tree that shares the maximum number of quartet topologies with input gene trees, restricting itself to a predefined set of bipartitions.

**Results:**

We introduce ASTRAL-III, which substantially improves the running time of ASTRAL-II and guarantees polynomial running time as a function of both the number of species (*n*) and the number of genes (*k*). ASTRAL-III limits the bipartition constraint set (*X*) to grow at most linearly with *n* and *k*. Moreover, it handles polytomies more efficiently than ASTRAL-II, exploits similarities between gene trees better, and uses several techniques to avoid searching parts of the search space that are mathematically guaranteed not to include the optimal tree. The asymptotic running time of ASTRAL-III in the presence of polytomies is $O\left ((nk)^{1.726} D \right)$ where *D*=*O*(*nk*) is the sum of degrees of all *unique* nodes in input trees. The running time improvements enable us to test whether contracting low support branches in gene trees improves the accuracy by reducing noise. In extensive simulations, we show that removing branches with *very* low support (e.g., below 10%) improves accuracy while overly aggressive filtering is harmful. We observe on a biological avian phylogenomic dataset of 14K genes that contracting low support branches greatly improve results.

**Conclusions:**

ASTRAL-III is a faster version of the ASTRAL method for phylogenetic reconstruction and can scale up to 10,000 species. With ASTRAL-III, low support branches can be removed, resulting in improved accuracy.

**Electronic supplementary material:**

The online version of this article (10.1186/s12859-018-2129-y) contains supplementary material, which is available to authorized users.

## Background

The potential for genome-wide discordance of evolutionary histories [1, 2] has motivated the development of several approaches for species phylogeny reconstruction. Reconstructing a collection of gene trees, each inferred from a different part of the genome, and then summarizing them to get a species tree is one such approach and is used by many phylogenomic projects (e.g., [3–7]) (while “gene trees” need not be inferred from functional genes, following the conventions of the field, we will refer to them as such). This two-step approach stands in contrast to concatenation [8], where all the data are combined and analyzed in a single analysis. The two-step approach aims to account for discordances between gene trees and the species tree (but its effectiveness is debated [9–12]) and is more computationally efficient than statistical co-estimation of gene trees and the species tree [13]. Incomplete lineage sorting (ILS) is a ubiquitous [14] cause of discordance. ILS is typically modeled by the multi-species coalescent model (MSCM) [15, 16], where branches of the species tree represent populations, and lineages are allowed to coalesce inside each branch; lineages that fail to coalesce at the root of each branch are moved to the parent branch.

Many “summary” methods have been developed to infer a species tree from a collection of input trees. Examples include MP-EST [17], NJst [18], ASTRID [19], DISTIQUE [20], ASTRAL [21, 22] and STAR [23], which only use gene tree topologies, and GLASS [24] and STEAC [23], which also use branch lengths. These methods are all proved statistically consistent under the MSCM, given error-free input gene trees; when input trees are inferred from sequence data, statistical consistency is not guaranteed [25]. Most methods take rooted gene trees as input, but some methods (e.g., ASTRAL, NJst/ASTRID and DISTIQUE) use unrooted input trees. ASTRAL-II [22] is currently one of the commonly used summary methods.

In this paper, we introduce an improved version of ASTRAL called ASTRAL-III. As we will show, compared to ASTRAL-II, the new version has better running time without sacrificing accuracy. The improvements in the running time are both theoretical (reducing the asymptotic running time so that it is guaranteed to grow polynomially with the dataset size) and empirical.

## Methods

### Notations and definitions

Let the set of *n* species be called *L* and let *G* be the set of *k* input gene trees on *L*. Let [*d*] represent the set {1,2…,*d*}. We use *Q*(*t*) to denote the set of quartet trees induced by a tree *t*. Any subset of *L* is called a cluster. We define a partition as a set of clusters that are pairwise mutually exclusive; note that we abuse the term “partition” here because the union of all clusters in a partition need not give the complete set. Each node in an unrooted tree defines a partition. A bipartition (tripartition) is a partition with cardinality two (three); a partition with cardinality at least four corresponds to a multifurcation (also referred to as a polytomy). Let *X* (the constraint bipartition set) be a set of clusters such that for each *A*∈*X*, we also have *L*−*A*∈*X*. We use *Y* to represent the set of all tripartitions that can be build from *X*:

$$\begin{aligned} Y=\{(A',A-A',L-A) : A' \subset A, A \in X, A' \in X, A-A' \in X\}. \end{aligned} $$

We use *E* to denote the set of all unique partitions and their frequency in *G*. Thus,

1 $$ E=\left\{\left(M, \sum_{g\in G}|N(g) \cap \{M\}|\right) : M\in N(g), g\in G\right\}  $$

where *N*(*g*) is the set of all partitions representing all internal nodes in the tree *g*. We also define *D* as the sum of the cardinalities of unique partitions in gene trees:

2 $$ D=\sum_{(M,c)\in E} |M|.  $$

### ASTRAL (old versions)

The problem addressed by ASTRAL is to find the tree that shares the maximum number of induced quartet topologies with the collection of input gene trees: Problem statement: Given a set *G* of input gene trees, find the species tree *t* that maximizes $\sum _{g \in G} |Q(g)\cap Q(t)|$.

Lafond and Scornavacca recently proved this problem is NP-hard [26].

#### ASTRAL-I and ASTRAL-II algorithms

ASTRAL solves a constrained version of the problem where a set of clusters *X* restricts bipartitions that the output species tree may include (recall ∀*A*∈*X*:*L*−*A*∈*X*). Note that setting *X* to the powerset solves the unconstrained problem. Based on the fact that an unrooted quartet species tree always matches the most likely unrooted quartet gene tree [27], ASTRAL is proved statistically consistent [21].

ASTRAL uses dynamic programming to solve the problem using the recursive relation:

$${{} \begin{aligned} V(A) &= \left\{\begin{array}{lc} 0 & |A|=1\\ \max_{A'\subset A, (A'|A-A'|L-A)\in Y} V(A,A') & |A|>1 \end{array}\right.\\ V(A,A') &= V(A') + V(A-A') + w(A'|A-A'|L-A) \end{aligned}} $$

where the function *w*(*T*) scores each tripartition *T*=(*A*|*B*|*C*) against each node in each input gene tree. Let partition $M=\left (M_{1}|M_{2}|...|M_{d}\right)$ represent an internal node of degree *d* in a gene tree. The overall contribution of *T* to the score of any species tree that includes *T* is:

3 $$ w(T)=\sum_{g\in G}\sum_{M\in N(g)}\frac{1}{2}QI(T,M)  $$

where, defining $a_{i}=|A\cap M_{i}|$, $b_{i}=|B\cap M_{i}|$, and $c_{i}=|C\cap M_{i}|$, we have:

4 $$  {{} \begin{aligned} QI(T, M)=\sum_{i\in [d]}\sum_{j\in [d]-\{i\}}\sum_{k\in [d]-\{i,j\}} \frac{a_{i}+b_{j}+c_{k} -3}{2} a_{i} b_{j} c_{k}. \end{aligned}}  $$

As previously proved [21], *QI*(*T,M*) computes twice the number of quartet trees that are going to be shared between any two trees if one includes only *T* and the other includes only *M*. ASTRAL-II requires $\Theta \left (d^{3}\right)$ time for computing *QI*(.), making its overall running time $O\left (n^{3}k|Y|\right)$ with polytomies of unbounded degrees or *O*(*nk*|*Y*|) in the absence of polytomies.

Noting trivially that |*Y*|<|*X*|^2^, the previously published running time analysis of ASTRAL-II was $O\left (nk|X|^{2}\right)$ for binary gene trees and $O\left (n^{3}k|X|^{2}\right)$ for trees with polytomies. A recent result by Kane and Tao [28] (motivated by the analysis of ASTRAL) proved that $\phantom {\dot {i}\!}|Y|\leq |X|^{3/log_{3}(27/4)}$. This result immediately gives us a better upper bound on the running time.

##### **Corollary 1**

ASTRAL-II runs in $O\left (nk|X|^{{1.726}}\right)$ and $O\left (n^{3}k|X|^{{1.726}}\right)$, respectively, with and without polytomies in gene trees.

In ASTRAL-I, *X* is the set of all bipartitions observed in input gene trees. While sufficient for statistical consistency and often for accuracy, under some conditions, this set *X* is too restrictive. To address this shortcoming, ASTRAL-II [22] uses several heuristics (see Additional file 1: Appendix A) and further expands the set *X*. Even though ATRAL-II tries to limit |*X*|, it does not provide any guarantees as to how it grows with *n* and *k*. In the worst case, |*X*| can grow exponentially, and thus, ASTRAL-II does not guarantee polynomial running time. The relatively high accuracy of ASTRAL-II has been shown in several simulations [20, 22, 29, 30] and it has been adopted by the community as one of the main methods used in phylogenomics. ASTRAL has the ability to compute branch lengths in coalescent units [2] and a measure of branch support called local posterior probability [31].

#### Limitations of ASTRAL-II

Several shortcomings of ASTRAL-II in terms of running time are addressed here (ASTRAL-III); our improvements, in turn, enable new types of analyses.

While ASTRAL-II can analyze datasets with a thousand species and gene trees in reasonable time, it does not easily scale to many tens of thousands of input trees. Datasets with more than ten thousand loci are already available (e.g., [5]) and as more genomes are sequenced, more are destined to become available in the near future. Moreover, being able to handle large *k* (i.e., numbers of input trees) enables using multiple trees per locus (e.g., a Bayesian sample) as input to ASTRAL. The limited scalability of ASTRAL with *k* has two reasons. First, the set *X* is not bounded in ASTRAL-II and can grow to become the power set. Thus, in ASTRAL-II, |*X*|*can* theoretically grow exponentially with *n*. We fix this in ASTRAL-III by modifying heuristics that form the set *X* so that they all guarantee that |*X*|=*O*(*nk*). The second cause of the slowdown is that computing each *w*(*T*) for a tripartition *T* requires *Θ*(*nk*). This computation does not exploit similarities between gene trees, a shortcoming that we fix in ASTRAL-III.

Beyond large *k*, ASTRAL-II, which scales as *O*(*n*^3^*k*|*X*|^1.726^) in the presence of polytomies, can quickly become prohibitively slow for input trees with large polytomies. ASTRAL-III uses a mathematical trick to enable scoring of gene tree polytomies in time similar to binary nodes. The ability to handle large polytomies in input gene trees is important for two reasons. Some of the conditions that are conducive to ILS, namely shallow trees, are also likely to produce identical gene sequence data for several species. The gene tree should leave the relationship between identical sequences unresolved (FastTree [32] automatically does it and RAxML, which outputs an arbitrary resolution, warns the user about the input). Moreover, all summary methods, including ASTRAL, are sensitive to gene tree estimation error [22, 33–37]. One way of dealing with gene tree error, previously studied in the context of minimizing deep coalescence [38], is to contract low support branches in gene trees and use these unresolved trees as input to the summary method. While earlier studies found no evidence that this approach helps ASTRAL-II when the support is judged by SH-like FastTree support [22], no study has tested this approach with bootstrap support values. We will for the first time evaluate the effectiveness of contracting branches with low *bootstrap* support and show that conservative filtering of *very* low support branches does, in fact, help the accuracy.

### ASTRAL-III

ASTRAL-III has six new features:

1. Heuristics for building the set *X* are modified to ensure |*X*|=*O*(*nk*). This step alone (without subsequent improvements) guarantees the overall running time is $O\left ((nk)^{2.726}\right)$ for binary gene trees and $O\left (n^{4.726}k^{2.726}\right)$ for polytomies.
2. Heuristics for building the set *X* are modified to enlarge *X* for gene trees with polytomies without breaking |*X*|=*O*(*nk*) guarantees. This can impact the accuracy and empirical running times but not the asymptotic running time.
3. A new way of computing *w*(*q*) is introduced to reduce the running time for scoring a gene tree to *O*(*n*), instead of $O\left (n^{3}\right)$, in the presence of polytomies. This step, combined with the previous steps, reduces the total running time to $O\left ((nk)^{2.726}\right)$ irrespective of whether gene trees have polytomies.
4. A polytree is used to represent gene trees, and this enables an algorithm that reduces the overall running time from $O\left ((nk)^{2.726}\right)$ to *O*(*D*.(*nk*)^1.726^), which is the final theoretical analysis of ATRAL-III running time.
5. A new algorithm, similar to A* [39], is used to compute an upper-bound on the best possible resolution of a clade; we need not expand a clade recursively when its upper-bound is below the best available score. The worst case asymptotic running time does not change due to this feature.
6. A two-stage heuristic mechanism is designed to further tighten the upper bounds used in pruning unnecessary parts of the search space. The worst case asymptotic running time is not impacted.

A beta version of ASTRAL-III was recently described [40] and that version included features 3–5 but not the others. We next describe each improvement.

#### New search space: |*X*|=*O*(*nk*)

ASTRAL-II uses several heuristic methods to build *X* (see the original paper [22] for details). The main method involves computing several extended majority consensus trees from gene trees and then resolving polytomies in these consensus trees using three techniques (mentioned below). These steps are repeated for 10 rounds or more until very few (less than a constant threshold) of the bipartitions observed are new to *X*. Because the number of rounds is not constant or a function of *n* and *k*, we cannot bound how *X* grows with *n* and *k* for ASTRAL-II. In ASTRAL-III, we limit the number of rounds by a constant value (default set to 110). This enables us to provide guarantees of a polynomial growth of |*X*| with *n* and *k*.

To get to *X*=*O*(*nk*), we need further changes. As mentioned, three techniques are used to resolve each polytomy of degree *d* in extended majority consensus trees. The first technique uses a precomputed distance matrix to build a UPGMA tree starting from sides of the polytomy and adds the new bipartitions from the UPGMA tree to *X*. This can only add *O*(*d*)=*O*(*n*) resolutions. The second technique computes a greedy consensus of gene trees subsampled to randomly selected taxa (one from each side of the polytomy) and adds bipartitions from the greedy consensus to *X*. This also can only add *O*(*d*) new bipartitions. The third resolution samples a taxon from each side of the polytomy, computes *d* caterpillar trees, each constructed based on decreasing similarity to each sampled taxon, and adds the bipartitions from all these caterpillar trees to *X*. This quadratic resolution step can add $O\left (d^{2}\right)=O\left (n^{2}\right)$ bipartitions to *X*. To have |*X*|=*O*(*n*), we need to change this step. Let *d*_1_…*d*_*r*_ be the list of all polytomy degrees in an extended majority consensus tree in the ascending ordered. We find the smallest threshold *q* such that $\sum _{i=1}^{q} d_{i}^{2} \leq c n$ for some constant *c* (default =25). In ASTRAL-III, we apply the quadratic resolution technique only for polytomies *d*_1_…*d*_*q*_; this, by definition, ensures no more than *O*(*d*)=*O*(*n*) bipartitions are added in each round.

#### New search space: handling gene tree polytomies

We also change the way ASTRAL builds *X* in the presence of gene tree polytomies. Our goal is to increase |*X*| compared to ASTRAL-II for multifurcating gene trees. However, |*X*| is enlarged at most by a constant factor and we retain |*X*|=*O*(*nk*).

If a gene tree includes polytomies, ASTRAL-II adds bipartitions implied by resolutions of that polytomy to the set *X* using a guide tree *g*. To build *g*, a greedy consensus of all gene trees is computed and is further refined to become binary by applying UPGMA to each polytomy of the greedy tree using a precomputed similarity matrix (see the original paper [22] for details). To resolve a gene tree polytomy of degree *d*, ASTRAL-II first randomly samples *d* taxa, each from one side of the polytomy. Let *S* be the sampled taxa. All bipartitions from the tree *g* restricted to the set *S* of leaves are added to *X*. While in ASTRAL-II this process is done only once, in ASTRAL-III, we repeat the process three times with different random samples *S*. This increases |*X*| but at most by a constant factor. The enlarged *X* can lead to improved accuracy when input trees include many polytomies.

The second change in ASTRAL-III is that we now use a UPGMA tree inferred based on the similarity matrix as the guide tree. We observed that the UPGMA tree summarizes the input gene trees more accurately than the greedy tree (see Additional file 1: Table S1). Finally, in ASTRAL-III, we improve the definition of the similarity matrix in the presence of gene tree polytomies. Unlike in ASTRAL-II, we ensure that unresolved quartet trees induced by gene trees do not increase the similarity between pairs of taxa included in those quartets. Note that the similarity matrix, which is based on quartets, should not be confused with the quartet score optimized by ASTRAL.

#### Efficient handling of Polytomies

Recall that ASTRAL-II uses Eq. 4 to score a tripartition against a polytomy of size *d* in *Θ*(*d*^3^) time. Our next Lemma shows that this can be improved.

##### **Lemma 1**

Let *QI*(*T,M*) be twice the number of quartet tree topologies shared between an unrooted tree that only includes a node corresponding to the tripartition *T*=(*A*|*B*|*C*) and another tree that includes only a node corresponding to a partition $M=\left (M_{1}|M_{2}|...|M_{d}\right)$ of degree *d*; then, *QI*(*T,M*) can be computed in time *Θ*(*d*).

##### *Proof*

In *Θ*(*d*) time, we can compute:

5 $$ S_{a} =\sum_{i\in [d]}a_{i}~~~\textmd{and}~~~ S_{a,b} =\sum_{i\in [d]}a_{i} b_{i}  $$

where $a_{i}=|A\cap M_{i}|$ and $b_{i}=|B\cap M_{i}|$; we can also compute *S*_*b*_, *S*_*c*_, *S*_*a,c*_ and *S*_*b,c*_ (similarly defined). Equation 4 computes twice the number of quartet tree topologies shared between an unrooted tree with internal node *T* and another tree with one internal node *M* [22]. Equation 4 can be rewritten as:

6 $$ \begin{aligned} QI\left((A|B|C), M\right)=&\sum_{i\in [d]}{{a_{i}}\choose{2}}\left((S_{b} - b_{i}) (S_{c} - c_{i}) - S_{b,c} + b_{i} c_{i}\right)\\ +&\sum_{i\in [d]}{{b_{i}}\choose{2}}\left((S_{a} - a_{i}) (S_{c} - c_{i}) - S_{a,c} + a_{i} c_{i}\right)\\ +&\sum_{i\in [d]}{{c_{i}}\choose{2}}\left((S_{a} - a_{i}) (S_{b} - b_{i}) - S_{a,b} + a_{i} b_{i}\right) \end{aligned}  $$

(the derivation is given in the Additional file 1: Appendix B). Computing Eq. 6 instead of Eq. 4 clearly reduces the running time to *Θ*(*d*) instead of $\Theta \left (d^{3}\right)$. □

ASTRAL needs to score each of the |*Y*| tripartitions considered in the dynamic programming against each internal node of each input gene tree. The sum of degrees of *k* trees on *n* leaves is *O*(*nk*) (since that sum can never exceed the number of bipartitions in gene trees) and thus:

##### **Corollary 2**

Scoring a tripartition (i.e., computing *w*) can be done in *O*(*nk*).

#### Gene trees as a Polytree

ASTRAL-II scores each dynamic programming tripartition against each individual node of each gene tree. However, nodes that are repeated in several gene trees need not be recomputed. Recalling the definitions of *E* and *D* (Eqs. 1 and 2),

##### **Lemma 2**

The score of a tripartition *T*=(*A*|*B*|*C*) against all gene trees (i.e., the *w*(*T*) score) can be computed in *Θ*(*D*).

##### *Proof*

In ASTRAL-III, we keep track of nodes that appear in multiple trees. This enables us to reduce the total calculation by using multiplicities:

7 $$ w(T)=\sum_{(M,c)\in E}c\times QI(T,M).  $$

We achieve this in two steps. In the first step, for each distinct gene tree cluster *W*, we compute the cardinality of the intersection of *W* and sets *A*, *B*, and *C* once using a depth-first search with memoization. Let *children*(*W*) denote the set of children of *W* in an arbitrarily chosen tree *g*∈*G* containing *W*. Then, we have the following recursive relation:

8 $$  |W \cap A| = \sum_{Z\in children(W)} |Z \cap A|  $$

(ditto for $|W \cap B|$ and $|W \cap C|$). All such intersection values can be computed in a post-order traversal of a polytree. In this polytree, all unique clusters in the gene trees are represented as vertices and parent-child relations are represented as edges; note that when a cluster has different resolutions in two different input trees, we arbitrary choose one set of children in building the polytree. The polytree will include no more than *D* edges; thus, the time complexity of traversing this polytree (to compute Eq. 8) for all nodes is *O*(*D*). Once all intersections are computed, in the second step, we simply compute the sum in Eq. 7. Each *QI*(.) computation requires *Θ*(*d*) by Lemma 1. Recalling that $D=\sum _{(M,c)\in E} |M|$, it is clear that computing Eq. 7 requires *Θ*(*D*). Therefore, both steps can be performed in *Θ*(*D*). □

##### **Theorem 1**

The running time of ASTRAL-III grows as $O\left (D(nk)^{{1.726}}\right)$ for both binary and multifurcating gene trees.

##### *Proof*

By results of Kane and Tao [28], the size of the set *Y* is $O\left (|X|^{{1.726}}\right)$, and for each element in *Y*, by Lemma 2, we require *O*(*D*) to compute the weights, regardless of the presence or absence of polytomies. The running time of ASTRAL is dominated by computing the weights [22]. Thus, the overall running time is $O(D|Y|)=O\left (D|X|^{1.726}\right)$. Moreover, ASTRAL-III forces |*X*| to grow as *O*(*nk*), giving the overall running time of $O\left (D(nk)^{1.726}\right)$ □

#### Trimming of the dynamic programming

We now introduce an upper-bound (proved in Additional file 1: Appendix B):

$$V(A) \leq U(A)=\frac{w(A|A|L)}{2}-\frac{w(A|A|A)}{3}. $$

Let *U*(*A,A*^″^)=*U*(*A*^″^)+*U*(*A*−*A*^″^)+*w*(*A*^″^|*A*−*A*^″^|*L*−*A*). Since *V*(*A*)≤*U*(*A*), for any (*A*^′^|*A*−*A*^′^|*L*−*A*^′^)∈*Y* and (*A*^″^|*A*−*A*^″^|*L*−*A*^″^)∈*Y*, we no longer need to recursively compute *V*(*A*^″^) and *V*(*A*−*A*^″^) when *U*(*A,A*^″^)≤*V*(*A,A*^′^). When computing *V*(*A*) by maximizing the score over all resolutions of *A*, imagine that we first encounter *A*^′^ and then *A*^″^. We avoid expanding *A*^″^ when *U*(*A,A*^″^)≤*V*(*A,A*^′^). This approach clearly makes the order of processing of the resolutions important. To heuristically improve the efficiency of this approach, we order all (*A*^′^|*A*−*A*^′^|*L*−*A*)∈*Y* according to *U*(*A,A*^′^). Note that computing *U*(*A*) does not require recursive computations down the dynamic programming DAG. Thus, the use of this upper-bound results in the trimming of the search space. However, as far as we can tell, this trimming does not improve the theoretical running time.

#### Two-staged *α*-trimming

In order to further trim the search space, another upper-bound of *V*(*A*) is calculated. For a given *α*≥1 and any ordering of the set $\left \{A' : (A'|A-A'|L-A) \in Y \right \}$ denoted by *A*_1_…*A*_*r*_, we define *V*_*α*_(*A*) as follows.

$$\begin{aligned} {V_{\alpha}}^{i}(A) &= \left\{ \begin{array}{lr} 0, & i = 0 \\ {V_{\alpha}}(A,A_{i}), & {V_{\alpha}}(A,A_{i}) > \alpha {V_{\alpha}}^{i-1}(A)\\ {V_{\alpha}}^{i-1}(A), & \text{otherwise} \end{array}\right\} ~~ \text{for} 0\leq i\leq r\\ {V_{\alpha}}(A,A_{i}) &= {V_{\alpha}}(A_{i})+{V_{\alpha}}(A-A_{i})+w(A_{i}|A-A_{i}|L-A) ~~ \text{and}\\& {V_{\alpha}}(A) = {V_{\alpha}}^{r}(A) \end{aligned} $$

We can compute *V*_*α*_(*A*) using an algorithm equivalent to our dynamic programming for computing *V*(*A*), except that, as resolutions of a clade *A* are being tested, a new one is accepted only if it improves upon the previous best resolution by a factor of *α* (thus, *α*=1 simply reproduces our existing dynamic programming). When computing *V*_*α*_(*A*), for any *i*<*j*, if $\alpha \left (V_{\alpha }(A,A_{i})\right) \geq U(A,A_{j})$, then it is guaranteed that $\alpha \left (V_{\alpha }(A,A_{i})\right) \geq V_{\alpha }\left (A,A_{j}\right)$, and thus we no longer need to recursively compute *V*_*α*_(*A*_*j*_) and *V*_*α*_(*A*−*A*_*j*_). After all *V*_*α*_(*A*) values are computed for some choice of *α*, we turn to computing *V*(*A*).

Observe that *V*_*α*_(*A*)≤*V*(*A*)≤*αV*_*α*_(*A*). Let *U*_*α*_(*A,A*_*j*_) be defined as

$$\begin{aligned} \min\left(U(A_{j}), \alpha V_{\alpha}(A_{j})\right) & + \min\left(U(A-A_{j}), \alpha V_{\alpha}(A-A_{j})\right) \\&\quad+ w(A_{j}|A-A_{j}|L-A) \end{aligned} $$

and note that *U*_*α*_(*A,A*_*j*_)≥*V*(*A,A*_*j*_)=*V*(*A*_*j*_)+*V*(*A*−*A*_*j*_)+*w*(*A*_*j*_|*A*−*A*_*j*_|*L*−*A*). Thus, during the dynamic programming, for *i*<*j*, if *V*(*A,A*_*i*_)>*U*_*α*_(*A,A*_*j*_), then it is guaranteed that *V*(*A,A*_*i*_)≥*V*(*A,A*_*j*_), and thus we no longer need to recursively compute *V*(*A*_*j*_) and *V*(*A*−*A*_*j*_). The hope is that the *U*_*α*_ function will give us tighter upper bounds compared to the *U* function previously defined. Whether this happens or not depends on the choice of *α*, the order of visiting clusters, and the particularities of a dataset.

While any choice of *α*≥1 would guarantee the correct solution to the dynamic programming, we have empirically selected a heuristic to choose *α*. We set $\alpha = \frac {U(L)}{g(L)}$, where *g*(*A*)=*g*(*A*_*i*_)+*g*(*A*−*A*_*i*_)+*w*(*A*_*i*_|*A*−*A*_*i*_|*L*−*A*) where *i*=arg max_*j*_*U*(*A*_*j*_)+*U*(*A*−*A*_*j*_)+*w*(*A*_*j*_|*A*−*A*_*j*_|*L*−*A*) and *g*(*A*)=0 for |*A*|=1. Just as before, we order the clusters in the decreasing order of *U*(*A,A*_*i*_).

## Results

### Experimental setup

We study three research questions: RQ1: Can *contracting low support branches* improve the accuracy of ASTRAL? RQ2: How do the *running time and search space* compare between ASTRAL-II and ASTRAL-III? RQ3: How *accurate* is ASTRAL-III, which guarantees polynomial size search space, compared to ATRAL-II?

#### Datasets

##### Avian biological dataset:

Neoavian relationships show extremely high levels of gene tree discord, perhaps because their ancestors experienced a rapid radiation [5]. A dataset of 48 genomes representing all avian orders has been used to partially resolve this rapid radiation [5]. A set of 14,446 loci (including exons, introns, and UCEs) was used to produce two reference species trees using concatenation and using a coalescent-based method [5, 33]. We use the set of all unbinned gene trees and compare ASTRAL-III with and without contraction against both reference trees.

##### Simulated avian-like dataset:

This simulated dataset, previously used to emulate the biological avian dataset [33], has three model conditions with respect to the simulated levels of ILS: 1X is the default, whereas 0.5X divides each branch length in half (increasing ILS) and 2X multiplies them by 2 (reducing ILS). Average RF distances between true species tree and true gene trees are 0.35, 0.47, and 0.59, respectively for 2X, 1X, and 0.5X. To further test the impact of gene tree estimation error, sequence lengths were also varied to create four model conditions: 250bp alignments (0.67 RF distance between true gene trees and estimated gene trees), 500bp (0.54 RF), 1000bp (0.39 RF) and 1500bp (0.30 RF), all based on the 1X ILS. We use 1000 gene trees, and 20 replicates per condition. Gene trees are estimated using RAxML [41] with 200 replicates of bootstrapping.

##### SimPhy-homogeneous (S100):

**Fig. 1:**
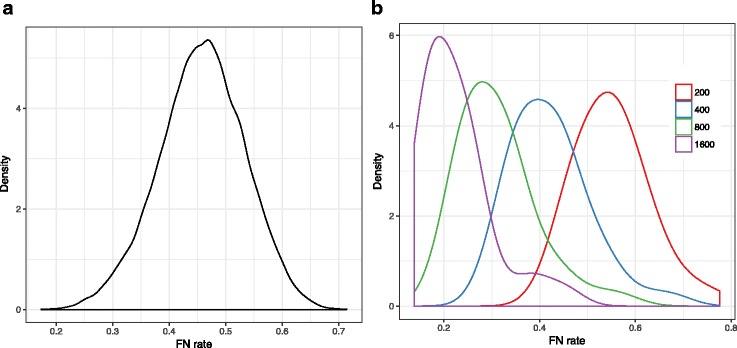
Properties of the S100 dataset. **a** The density plot of the amount of true gene discordance measured by the FN rate between the true species tree and the true gene trees. **b** The density plot of gene tree estimation error measured by FN rate between true gene trees and estimated gene trees for different sets of sequence lengths

We simulated a new 101-taxon dataset using SimPhy [42] with 50 replicates, each with a different species tree. The species trees are simulated under the birth-only process with birth rate 10^−7^, fixed haploid *N*_*e*_ of 400K, and the number of generations sampled from a log-normal distribution with mean 2.5M. For each replicate, 1000 true gene trees are simulated under the MSCM (exact commands shown in Additional file 1: Appendix C and parameters given in Additional file 1: Table S2). The average normalized RF distance between true species trees and true gene trees was in most replicates in the $\left [0.3,0.6\right ]$ range, with an average of 0.46 (Fig. 1). We use Indelible [43] to simulate the nucleotide sequences along the gene trees using the GTR evolutionary model [44] with 4 different fixed sequence lengths: 1600, 800, 400, and 200bp. We then use FastTree2 [32] to estimate both ML and 100 bootstrapped gene trees under the GTR+ *Γ* (requiring more than two million runs in total). Gene tree estimation error, measured by the FN rate between the true gene trees and the estimated gene trees, depended on the sequence length as shown in Fig. 1 (0.55, 0.42, 0.31, and 0.23 on average for 200bp, 400bp, 800bp, and 1600bp, respectively). We sample 1000, 500, 200, or 50 genes to generate datasets with varying numbers of gene trees.

##### SimPhy-ASTRAL2 (S200):

This dataset (201 taxa) is from the ASTRAL-II paper [22]. We use its most challenging model conditions with max tree height set to 500K generations and two rates of speciation: 10^−6^ and 10^−7^ (respectively, recent and deep speciation events). Compared to S100, this dataset has a much higher level of ILS. This was the only case in the ASTRAL-II paper where enlarging *X* substantially impacted accuracy [22]. We use S200 to test if our changes to *X* have compromised the accuracy. Like S100, gene alignments have varying lengths and mutation rates, leading to a wide range of gene tree error [22]. We analyze the data using 1000, 200, or 50 genes, and each model condition has 50 replicates; following the original paper, three replicates with low signal are removed.

#### Methods and Evaluation

We compare ASTRAL-III (version 5.5.4) to ASTRAL-II (version 4.11.1) in terms of running time and accuracy. To address RQ1, we draw bootstrap support values on the ML gene trees and then contract branches with bootstrap support up to a threshold (0, 3, 5, 7, 10, 20, 33, 50, and 75%,) using the newick utility package [45]. Together with the original gene trees, we have 10 different versions of ASTRAL-III.

To measure the accuracy of estimated species trees, we use False Negative (FN) rate. Note that in all our species tree comparisons, FN rate is equivalent to normalized Robinson–Foulds (RF) [46] metric because the ASTRAL species trees are fully resolved. All running times are measured on a cluster with servers with Intel(R) Xeon(R) CPU E5-2680 v3 @ 2.50GHz; each run was assigned to a single process, sharing cache and memory with other jobs.

### RQ1: Impact of contracting low support branches on accuracy

We investigate RQ1 on the two simulated datasets where bootstrapping was feasible (avian and S100) and on the real avian dataset. On S200, due to its size, bootstrapping was not feasible and thus we cannot test RQ1.

#### S100

**Fig. 2:**
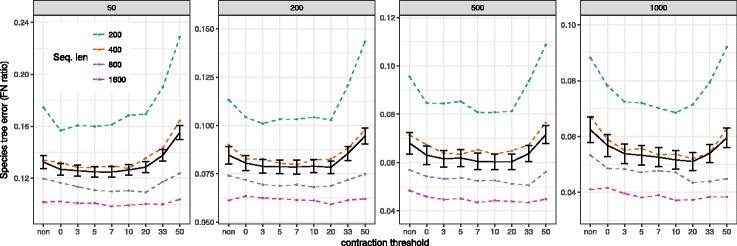
Impact of contraction on the S100 dataset. The FN error of ASTRAL-III species trees is shown on the S100 dataset given *k*= 50, 200, 500, or 1000 genes (*boxes*) run on the original FastTree gene trees (*non*) or gene trees with branches with ≤{0,3,5,7,10,20,33,50}% support contracted (*x-axis*). Average FN error and standard error bars (200 replicates) are shown with the four alignment lengths combined (*black solid line*). average FN error broken down by alignment length (50 replicates) is also shown (*dashed colored lines*)

On this dataset, contracting *very* low support branches in most cases improves the accuracy (Fig. 2 and Additional file 1: Table S3). However, the excessive removal of branches with high, moderate, or occasionally low support degrades the accuracy. Nevertheless, filtering at 10% is always beneficial on average (Additional file 1: Table S3). The threshold where contracting starts to become detrimental depends on the condition, especially the number of gene trees and the alignment length, perhaps representing a signal to noise ratio trade-off.

As the number of genes increases, the optimal threshold for contracting also tends to increase. Combining all model conditions, the error continues to drop until a 20% contracting threshold with 1000 genes, whereas no substantial improvement is observed after contracting branches with 5% support for 50 genes (Fig. 2). Nevertheless, removing branches with 10% or 20% does not increase the error with 50 genes. Perhaps, with few gene trees, removing branches of low support leaves us with very little information left; thus, regardless of whether we contract or not, we don’t get much signal around the most difficult branches. In contrast, when many gene trees are given, perhaps even after removing many branches, still enough gene trees with a resolution around difficult species tree branches are left.

The alignment length and gene tree error also impact the effect of contraction. For short alignments (200bp) and 1000 genes, contracting branches with up to 10% support reduces the species tree error by 21% (from 8.8% with no contraction to 6.9%). As alignment length grows, benefits of gene tree contraction diminish, so that with 1600bp genes, the reduction in error is merely from 4.1 to 3.7%. This pattern is perhaps expected because, with longer alignments, branch support is expected to increase. Thus, with longer gene alignments and consequently better gene trees with higher support, there is less room for improvement by reducing the noise. Consistent with this explanation, grouping replicates based on average gene tree error gives similar results as grouping by alignment length (see Additional file 1: Figure S1).

#### avian-like simulations

**Fig. 3:**
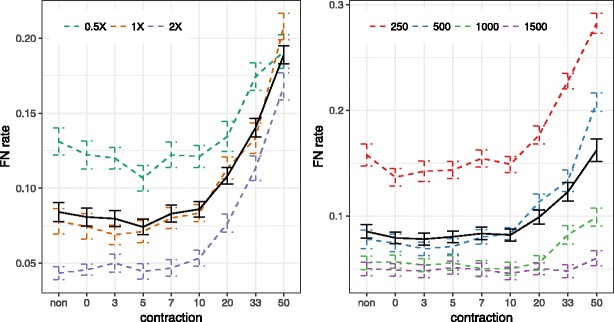
Impact of contraction on the avian simulated dataset. The FN error of ASTRAL-III species trees is shown on the avian simulated dataset given *k*=1000 genes with (*left*) fixed sequence lengths =500 and varying levels of ILS, or (*right*) fixed ILS (1X) and varying sequence length, in each case both with full FastTree gene trees (*non*) or trees with branches with ≤{0,3,5,7,10,20,33,50}% support contracted (*x-axis*). Average and standard error bars are shown for all conditions combined (*black solid line*) and also for each model condition separately (*dashed color lines*). Each model condition has 20 replicates

On the avian simulated dataset, contracting low support branches helps accuracy marginally, but the extent of impact depends on the model condition (Fig. 3). With moderate ILS (2X), we see no improvements as a result of contracting low support branches, perhaps because the average error is below 5% even with no contraction, leaving little room for improvements. Increasing ILS, we start to see improvements using contracted gene trees. Removing branches of up to 5% support reduces the error from 13 to 11% with 0.5X, and from 8 to 7% for the 1X condition.

When ILS is fixed to 1X and sequence length is varied (Fig. 3), contracting is helpful mostly with short sequences (e.g., 250 bp). With longer sequences, where gene tree estimation error is low, little or no improvement in accuracy is obtained. The best accuracy is typically observed by contracting at 0–5%. The gains in accuracy comparing no contraction to contraction at 0, 3, 5% thresholds are statistically significant (*p*=0.017, 0.028, and 0.013) according to one-tailed paired t-tests.

#### Avian biological dataset

**Fig. 4:**
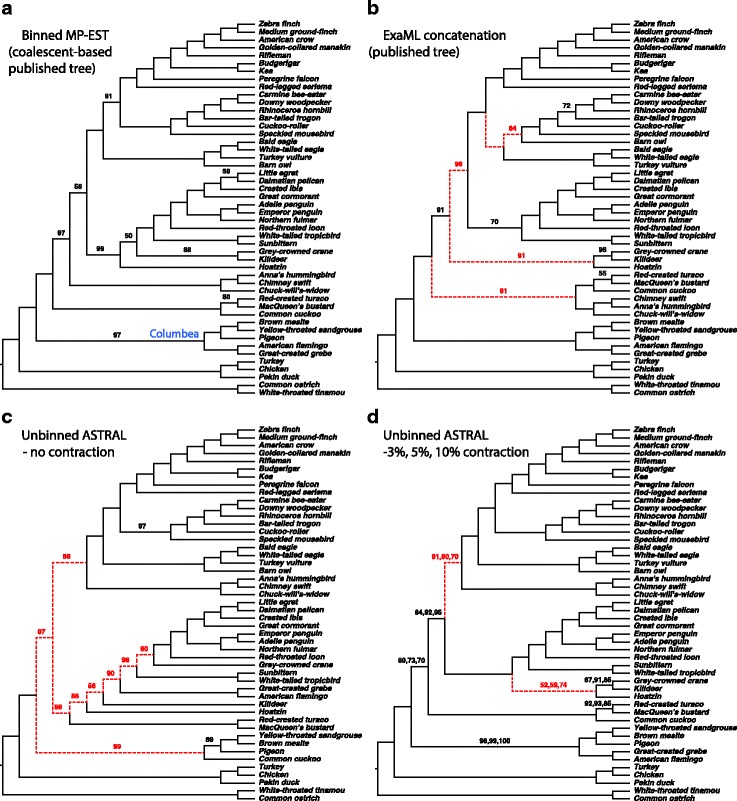
Avian dataset with 14,446 genes. Shown are reference trees from the original paper [5] using the coalescent-based binning (**a**) and concatenation (**b**), and two new trees using ASTRAL-III with no contraction (**c**) and with contraction with 3, 5, and 10% thresholds (**d**). Support values (bootstrap for **a**, **b** and local posterior probability for **c**, **d**) shown for all branches except those with full support; in (**d**), support is shown for 3, 5, and 10%, respectively. Branches conflicting with the reference coalescent-based tree are shown as dotted red lines

The original analyses on this dataset [5, 33] report that MP-EST [17] run on 14,446 gene trees produces a tree that conflicts with strong evidence from the literature and other analyses on the same dataset. The statistical binning method was developed to address this shortcoming by combining loci together to reduce gene tree error [33, 34]. MP-EST run on binned gene trees (i.e., binned MP-EST) produced results [5, 33] that were largely congruent with the concatenation using ExaML [47] and differed in only five branches with low support (Fig. 4a, b); both trees were used as the reference [5]. Here, we test if simply contracting low support gene tree branches and using ASTRAL-III produces trees congruent with the reference trees.

Similar to MP-EST, when ATRAL-III is run on 14,446 gene trees with no contraction, the results differ in nine and 11 branches, respectively, with respect to the reference binned MP-EST and concatenation trees (Fig. 4c). Moreover, this tree contradicts some strong results from the avian analyses (e.g., not recovering the Columbea group [5]). ASTRAL-III with no contraction finishes in 32 hours, but with contraction, depending on the threshold, it takes 3 to 84 h (>50 h for 0 – 20% thresholds and <26 hours for 33 – 75%). Contracting 0% branches has minimal impact on the discordance (eight discordant branches with binned MP-EST instead of nine). However, contracting low support branches with 3–33% thresholds dramatically reduces the discordance with the reference tree (2, 2, 4, 2, 3, and 3 discordant branches, respectively, for 3, 5, 7, 10, 20, and 33%). Three thresholds (3, 5, and 10%) produce an identical tree (Fig. 4d). The remaining differences are among the branches that are deemed unresolved by Jarvis *et al.* and change among the reference trees as well [5]. Contracting at 50 and 75% thresholds, however, increases discordance to five and six branches, respectively.

Thus, consistent with simulations, contracting very low support branches seems to produce the best results, when judged by similarity with the reference trees. To summarize, ASTRAL-III obtained on unbinned but collapsed gene trees agreed with all major relations in Jarvis et al., including the novel Columbea group, whereas the unresolved tree missed important clades (Fig. 4).

### RQ2: Running time improvements

#### Varying the number of genes (*k*)

**Fig. 5:**
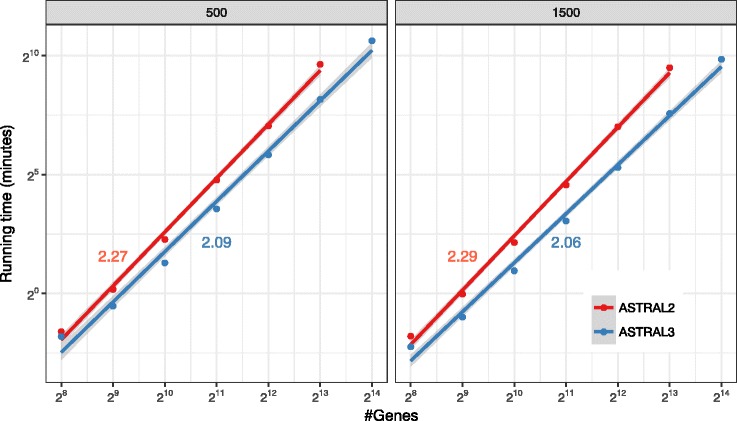
Running time versus *k*. Average running times (4 replicates) are shown for ASTRAL-II and ASTRAL-III on the avian dataset with 500bp or 1500bp alignments with varying numbers of gens (*k*), shown in log scale (see Additional file 1: Figure S2 for normal scale). A line is fit to the data points in the log/log space and line slopes are shown. ASTRAL-II did not finish on 2^14^ genes in 48 hours

We compare ASTRAL-III to ASTRAL-II on the avian simulated dataset, changing the number of genes from 2^8^ to 2^14^ and forcing *X* to be the same for both versions to enable comparing impacts of improved weight calculation (Fig. 5). We allow each replicate run to take up to two days. ASTRAL-III improves the running time over ASTRAL-II and the extent of the improvement depends on *k* (see Additional file 1: Figure S2). With 1000 genes or more, there is at least a 2.1X improvement. With 2^13^ genes, the largest value where both versions could run, ASTRAL-III finishes on average 3.2 times faster than ASTRAL-II (234 versus 758 minutes). ASTRAL-II is not able to finish on the dataset with *k*=2^14^, while ASTRAL-III finishes on all conditions. Moreover, fitting a line to the average running time in the log-log scale graph reveals that on this dataset, the running time of ASTRAL-III on average grows as *O*(*k*^2.08^), which is better than that of ASTRAL-II at *O*(*k*^2.28^), and both are better than the theoretical worst case, which is *O*(*k*^2.726^). These results are consistent with the fact that ASTRAL-III considers similarities between gene tree nodes.

#### Running time for large polytomies

**Fig. 6:**
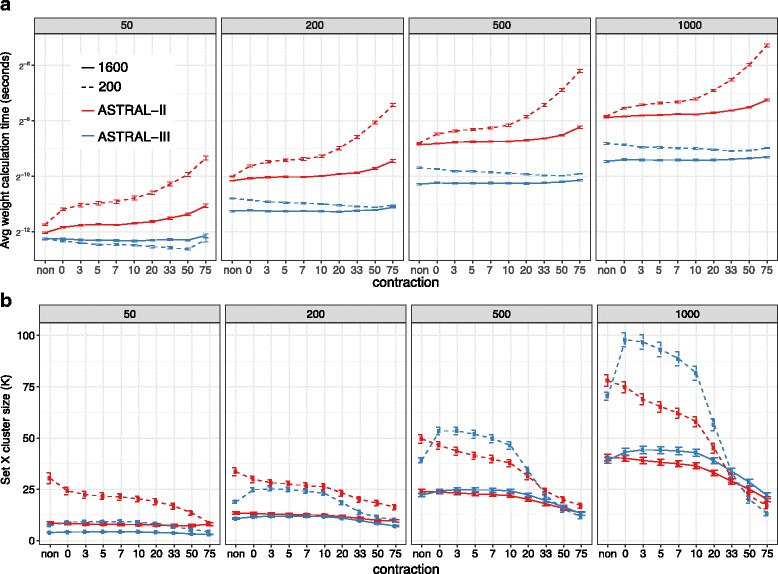
Weight calculation and |*X*| on S100. Average and standard error of (**a**) the time it takes to score a single tripartition using Eq. 3 and (**b**) search space size |*X*| are shown for both ASTRAL-II and ASTRAL-III on the S100 dataset. Running time is in log scale. We vary numbers of gene trees (*boxes*) and sequence length (200 and 1600). See Additional file 1: Figure S3 for similar patterns for with 400 and 800bp alignments

ASTRAL-III has a clear advantage compared to ASTRAL-II with respect to the running time when gene trees include polytomies (Fig. 6a and Additional file 1: Figure S3). Since ASTRAL-II and ASTRAL-III can have a different set *X*, we show the running time per each weight calculation (i.e., Eq. 3). As we contract low support branches and hence increase the prevalence of polytomies, the weight calculation time quickly grows for ASTRAL-II, whereas, in ASTRAL-III, the weight calculation time remains flat, or even decreases. These results are consistent with a change of asymptotic running time to score a polytomy of size *d* from *O*(*d*^3^) in ASTRAL-II to *O*(*d*) in ASTRAL-III.

#### The search space

Comparing the size of the search space (|*X*|) between ATRAL-II and ASTRAL-III shows that as intended, the search space is decreased in size for cases with no polytomy but can increase in the presence of polytomies (Fig. 6b). With no contraction, on average, |*X*| is always smaller for ASTRAL-III than ASTRAL-II. With few error-prone gene trees (50 gene trees from 200bp alignments), the search space has reduced dramatically but with many genes or high-quality gene trees, the reductions are minimal. Moreover, the search space for gene trees estimated from short alignments (e.g., 200 bp) is several times larger than those based on longer alignments (e.g., 1600 bp) for both methods. These are results of the first feature of ASTRAL-III that forces the search space to grow at *O*(*nk*).

Contracting low support branches initially increases the search space. This is because ASTRAL-III unlike ASTRAL-II adds multiple resolutions per polytomy to *X*. Further contraction results in reductions in |*X*|, presumably because many polytomies exist and they are resolved similarly inside ASTRAL-III.

### RQ3: ASTRAL-II versus ASTRAL-III accuracy

Despite limiting |*X*| to grow at most linearly with *n* and *k*, the accuracy of ASTRAL-III remains unchanged compared to ASTRAL-II (Table 1 and Additional file 1: Figures S4–S7). Importantly, even for the very challenging S200 dataset, the accuracy is not reduced substantially even though |*X*| is reduced by up to 47%. Over all datasets, differences in error are less than 0.002, except for three datasets where the error of ASTRAL-III was less than ASTRAL-II by 0.003, 0.005, and 0.006 and two cases where the error increased by 0.004. Over all datasets, the differences between ASTRAL-II and ASTRAL-III were not statistically significant according to a paired t-test (*p*-value = 0.496). Since ASTRAL-III has a reduced search space, its quartet scores are typically slightly lower than ASTRAL-II, but these reductions are never more than 0.06%. As expected, the largest drops in the quartet score happen for the challenging S200 dataset with only 50 gene trees. The search space reduces in almost all cases and the reductions can be as much as 72%. Thus, the improved running time of ASTRAL-III does not come at the price of reduced accuracy.

**Table 1:** ASTRAL-II versus ASTRAL-III. Average and standard error (inside parenthesis) are shown for changes in accuracy (normalized FN rate), quartet score, and search space size (|*X*|)

Data set	Model condition	FN	|*X*|	Quartet score
avian	0.5X-500bp	−0.006 (0.007)	−3% (0)	−0.01% (0.01)
	1X-1000bp	0.001 (0.002)	−1% (0)	0.00% (0.00)
	1X-1500bp	0.004 (0.003)	−1% (0)	0.00% (0.00)
	1X-250bp	0.004 (0.007)	−3% (0)	−0.01% (0.00)
	1X-500bp	−0.001 (0.004)	−2% (0)	0.00% (0.00)
	2X-500bp	−0.003 (0.003)	−2% (0)	0.00% (0.00)
S200	1000gt- 10^−6^	−0.001 (0.000)	0% (0)	0.00% (0.00)
	200gt- 10^−6^	0.000 (0.001)	−5% (1)	0.00% (0.00)
	50gt- 10^−6^	−0.001 (0.001)	−42% (2)	−0.06% (0.01)
	1000gt- 10^−7^	0.001 (0.001)	−1% (0)	0.00% (0.00)
	200gt- 10^−7^	−0.001 (0.001)	−6% (1)	0.00% (0.01)
	50gt- 10^−7^	0.000 (0.002)	−47% (2)	−0.06% (0.01)
S100	1000gt-1600bp	0.000 (0.000)	−3% (0)	0.00% (0.00)
	500gt-1600bp	0.000 (0.000)	−6% (1)	0.00% (0.00)
	200gt-1600bp	0.000 (0.001)	−17% (1)	−0.01% (0.00)
	50gt-1600bp	−0.001 (0.001)	−46% (3)	−0.01% (0.01)
	1000gt-200bp	−0.001 (0.002)	−9% (1)	0.00% (0.00)
	500gt-200bp	−0.001 (0.001)	−19% (1)	−0.01% (0.01)
	200gt-200bp	−0.001 (0.001)	−40% (1)	−0.01% (0.00)
	50gt-200bp	−0.002 (0.002)	−72% (1)	−0.05% (0.01)
	1000gt-400bp	−0.001 (0.002)	−6% (1)	0.00% (0.00)
	500gt-400bp	0.001 (0.001)	−12% (1)	−0.01% (0.00)
	200gt-400bp	0.000 (0.001)	−29% (2)	−0.01% (0.01)
	50gt-400bp	−0.005 (0.001)	−61% (2)	−0.02% (0.01)
	1000gt-800bp	0.000 (0.000)	−4% (0)	0.00% (0.00)
	500gt-800bp	0.001 (0.001)	−9% (1)	0.00% (0.00)
	200gt-800bp	0.001 (0.000)	−22% (2)	−0.01% (0.01)
	50gt-800bp	0.000 (0.001)	−52% (3)	−0.02% (0.01)

FN: we show ASTRALIII−ASTRALII; negative numbers indicate ASTRALIII is more accurate. |*X*|: we show $\frac {\text {ASTRALIII}-\text {ASTRALII}}{\text {ASTRALII}}\times 100$; negative numbers indicate that ASTRAL-III has a reduced search space. Quartet score: we show $\frac {\text {ASTRALIII}-\text {ASTRALII}}{\text {ASTRALII}}\times 100$; positive numbers indicate that ASTRALIII has improved quartet scores. See Additional file 1: Figures S4–S7 for full distributions

## Discussion

Below we further comment on ASTRAL-III in terms of accuracy and running time. We finish by comparing ASTRAL-III and ASTRAL-III-beta.

### Accuracy

Although tree accuracy can improve with contracted gene trees, the gap between performance on true gene trees and estimated gene trees remains wide (Additional file 1: Table S3). On the S100 dataset, respectively for 50, 200, 500, and 1000 genes, the best average error with 1600bp gene trees among all contraction levels were 9.8%, 5.9%, 4.3%, and 3.7% compared to 7.0%, 3.7%, 2.4%, and 1.5% with true gene trees. Thus, while contracting low support branches helps in addressing gene tree error, improved methods of gene tree estimation remain crucial. Our results also indicate that in the presence of noisy gene trees, increased numbers of genes are needed to achieve high accuracy. For example, on the S100 dataset, with 1000 gene trees of only 200bp and contracting with a 10% threshold, the species tree error was 6.9%, which slightly outperformed the accuracy with only 50 true gene trees. This observation encourages the use of a large number of gene trees; incidentally, a main feature of ASTRAL-III is improved running time with many genes.

The best choice of the threshold of contraction was somewhat sensitive to the dataset. Testing up to 1000 gene trees, we observed that more gene trees clearly increased the optimal threshold, but did not test beyond 1000 genes. One can predict that perhaps the trend may continue but also that the optimal threshold will not indefinitely increase. Similarly, we saw that the amount of gene tree error due to lack of signal impacts the optimal threshold. One may expect that other sources of error, including incorrect orthology, incorrect alignment, and model misspecifications may also impact the optimal threshold. Regardless of the choice of the optimal threshold, it seems that the largest benefits are associated with removing the least supported branches. Overall, a threshold of 10% seemed to provide a good default value.

In most datasets, a substantial accuracy improvement was observed when 0% BS branches were removed. Branches of 0% support are presumably resolved arbitrarily. The use of conserved genes or closely related taxa can increase instances where two or more taxa have identical sequences in some genes. Some tree estimation methods generate binary trees even under such conditions. Removing branches that are arbitrarily resolved make sense and, as our results indicate, improves accuracy.

The main competitor of ASTRAL is NJst [18] and its fast implementation, ASTRID [19], but these tools are not able to handle polytomies in input gene trees. ASTRAL-III makes it efficient to use unresolved gene trees. Moreover, beyond contracting low support branches, other strategies could be used to reduce impacts of gene tree uncertainty. Previous studies indicate that simply using the set of all bootstrap gene tree replicates as input to ASTRAL increases error [21], perhaps due to the increased noise [31, 35]. However, using a sample from the Bayesian distribution for each gene tree may improve the accuracy of ASTRAL.

Finally, theoretical implications of removing low support branches are less clear than its empirical impact. In principle, branches that have low support are not necessarily expected to be randomly selected among gene trees. Thus, while our empirical results support the use of (conservative) filtering, the resulting procedure may lose statistical guarantees of consistency. Future work should study conditions where ASTRAL remains statistically consistent with contracted gene trees.

### Running time

#### Large *n*

To assess limits of ASTRAL-III in terms of scalability, we tested it on 20 replicates of a dataset with 5,000 species and 1000 true gene trees (simulation procedure described in Additional file 1: Appendix C and parameters given in Additional file 1: Table S4). ASTRAL-III took between 2 and 62 h to run on this dataset (9.4 hours on average). We also attempted to test ASTRAL-III on four replicates of a dataset with 10,000 species and 1000 true gene trees, allowing a week of running time. Of the four replicates, two were able to finish within the allotted time. Thus, depending on the nature of the data, ASTRAL-III may be able to scale to datasets with up to 10,000 species given sufficient running time.

#### Average running time, |*X*|, and |*Y*|

The ASTRAL-III running time analysis is based on several worst-case assumptions, and real data may grow less rapidly with both *n* and *k*. Overall, although the exact value depends on the dataset and especially the amount of discordance, the running time of ASTRAL seems to grow roughly quadratically with both *n* and *k* (i.e., proportionally to *n*^2^*k*^2^); see Additional file 1: Figures S2 and S8.

**Fig. 7:**
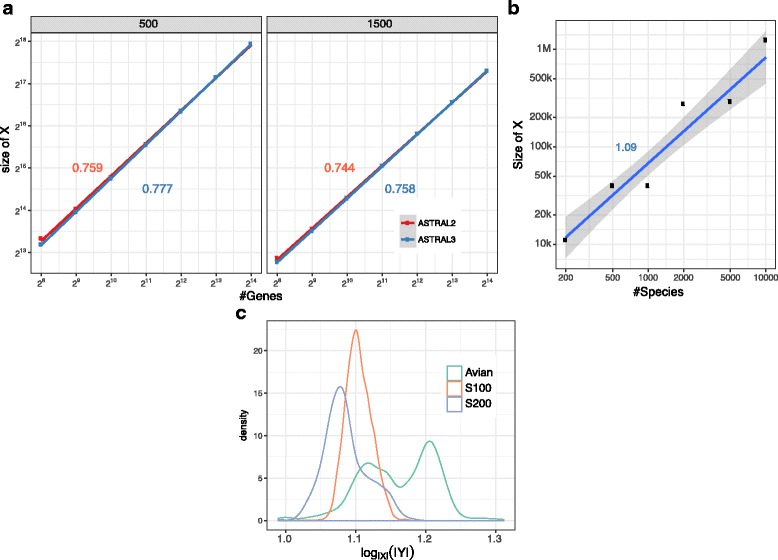
Empirical search space. **a** |*X*| is shown for ASTRAL-II and ASTRAL-III for avian-like simulateds dataset with varying numbers of genes. **b** |*X*| is shown for ASTRAL-III for several datasets with varying *n*. **c** The density plots of $\log _{X} |Y|$ across all ASTRAL-III runs reported in this paper. Size of the dynamic programming space *Y* is never above |*X*|^1.312^ here

ASTRAL-III bounds |*X*| to grow at most linearly with *n* and *k*. Empirically, we observe that |*X*| grows sublinearly with *k*$\left (\mathrm {close to} O\left (k^{\frac {3}{4}}\right)\right)$ on the avian simulated dataset (Fig. 7a). Note that the avian dataset has one of the highest levels of ILS; the dependence on *k* is expected to be lower for datasets with lower gene tree discordance. Testing the growth with *n* is more difficult because as *n* changes, other factors such as the amount of discordance also change. Nevertheless, across all the datasets that we had available, we tested the change in running time for fixed *k* as *n* changes and observed a linear growth (Fig. 7b), matching the worst-case scenario.

Finally, establishing empirical running time growth requires establishing the rate of the growth of |*Y*| with respect to |*X*|. The |*Y*|≤|*X*|^1.726^ upper-bound is for specialized formations of the set *X* [28]. Empirically, as |*X*| increases, the size of |*Y*| in ASTRAL-III does not increase as fast as the worst-case scenario implies. Across all of our ASTRAL-III runs in this paper, |*Y*| ranged in 90% of our runs between |*X*|^1.07^ and |*X*|^1.20^, and the overall average was |*X*|^1.11^ (Fig. 7c).

### Comparisons to ASTRAL-III-beta

The beta version of ASTRAL-III [40] included features 3–5 but not changes to *X* (features 1 and 2) or the two-staged *α*-trimming technique (feature 6). For completeness, we compared ASTRAL-III-beta and ASTRAL-III in terms of accuracy, quartet score, and the running time (Table 2). Accuracy and quartet scores are very similar, perhaps with a small improvement since the beta version. The search space is reduced since the beta version (due to features 1 and 2), and the running times are substantially decreased (at least by half in most cases). The reductions in the running time are due to *α*-trimming, reduced |*X*|, in addition to further improvements in details of our implementation of the polytree data-structure.

**Table 2:** ASTRAL-III-beta vs ASTRAL-III. Columns are defined similar to Table 1.

Model	Contraction	FN	|*X*|	|*Y*|	Quartet	Running
condition					score	time
avian-0.5X-500bp	None	−0.003	−3%	−9%	−0.02%	−48%
avian-1X-250bp	None	−0.001	−3%	−9%	0.00%	−56%
avian-1X-500bp	None	−0.001	−2%	−6%	0.00%	−50%
avian-1X-1000bp	None	−0.001	−1%	−4%	0.00%	−58%
avian-1X-1500bp	None	0.001	−1%	−4%	0.00%	−57%
avian-2X-500bp	None	−0.002	−2%	−4%	0.00%	−65%
avian-0.5X-500bp	10%	−0.003	−3%	−29%	−0.01%	−69%
avian-1X-250bp	10%	−0.001	−50%	−40%	0.00%	−81%
avian-1X-500bp	10%	0.003	−18%	−62%	−0.01%	−62%
avian-1X-1000bp	10%	0.000	−5%	−8%	0.00%	−61%
avian-1X-1500bp	10%	0.003	0%	−1%	0.00%	−55%
avian-2X-500bp	10%	−0.002	−14%	−18%	0.00%	−62%

Negative numbers indicate ASTRAL-III-beta has a larger value (i.e., has higher error, larger search space, better quartet scores, and is slower)

To further demonstrate the impact of the *α*-trimming feature, we randomly chose 18 species from the avian dataset with 1500bp and 1X ILS. On this limited dataset, we ran ASTRAL-III in its exact mode (i.e., setting *X* to the power set) with 100 gene trees. Without any trimming of the dynamic programming (i.e., without features 5 and 6), the running time was 40 minutes. Emulating ASTRAL-III-beta, we disabled *α*-trimming but kept the trimming (feature 5) and the running time reduced to 33 min. Adding the *α*-trimming feature dramatically reduced the running time to 13 min. Thus, when *X* includes many bipartitions that have very little promise in improving the quartet score (as in the exact mode of ASTRAL), the *α*-trimming approach is very effective in reducing the running time.

## Conclusions

We introduced ASTRAL-III, which compared to ASTRAL-II, improves scalability, especially for datasets with large *k* and many polytomies. These improvements enabled us to test the accuracy of ASTRAL after contracting low support branches. Overall, we observed improvements in accuracy when very low support branches were contracted, but also evidence that aggressive filtering reduces the accuracy. ASTRAL-III bounds the theoretical running time to $O\left ((nk)^{{1.726}}.D\right)$ where *D*=*O*(*nk*) is the sum of degrees of all unique gene tree nodes. In practice, the running time tends to grow no worse than quadratically with both *n* and *k*.

## Additional file

Additional file 1 Supplementary material and appendices. Appendices A, B, and C in addition to **Figures S1–S8**, and **Tables S1–S4** are all provided as one Additional file 1. (PDF 476 kb)

## References

[1] Maddison WP (1997). Gene trees in species trees. Syst Biol.

[2] Degnan JH, Rosenberg NA (2009). Gene tree discordance, phylogenetic inference and the multispecies coalescent. Trends Ecol Evol.

[3] Song S, Liu L, Edwards SV, Wu S (2012). Resolving conflict in eutherian mammal phylogeny using phylogenomics and the multispecies coalescent model. Proc Natl Acad Sci.

[4] Wickett NJ, Mirarab S, Nguyen N, Warnow T, Carpenter EJ, Matasci N, Ayyampalayam S, Barker MS, Burleigh JG, Gitzendanner MA, Ruhfel BR, Wafula E, Der JP, Graham SW, Mathews S, Melkonian M, Soltis DE, Soltis PS, Miles NW, Rothfels CJ, Pokorny L, Shaw AJ, DeGironimo L, Stevenson DW, Surek B, Villarreal JC, Roure B, Philippe H, DePamphilis CW, Chen T, Deyholos MK, Baucom RS, Kutchan TM, Augustin MM, Wang J, Zhang Y, Tian Z, Yan Z, Wu X, Sun X, Wong GK-S, Leebens-Mack J (2014). Phylotranscriptomic analysis of the origin and early diversification of land plants. Proc Natl Acad Sci.

[5] Jarvis ED, Mirarab S, Aberer AJ, Li B, Houde P, Li C, Ho SYW, Faircloth BC, Nabholz B, Howard JT, Suh A, Weber CC, da Fonseca RR, Li J, Zhang F, Li H, Zhou L, Narula N, Liu L, Ganapathy G, Boussau B, Bayzid MS, Zavidovych V, Subramanian S, Gabaldón T, Capella-Gutiérrez S, Huerta-Cepas J, Rekepalli B, Munch K, Schierup MH, Lindow B, Warren WC, Ray D, Green RE, Bruford MW, Zhan X, Dixon A, Li S, Li N, Huang Y, Derryberry EP, Bertelsen MF, Sheldon FH, Brumfield RT, Mello CV, Lovell PV, Wirthlin M, Schneider MPC, Prosdocimi F, Samaniego JA, Velazquez AMV, Alfaro-Núñez A, Campos PF, Petersen B, Sicheritz-Ponten T, Pas A, Bailey T, Scofield P, Bunce M, Lambert DM, Zhou Q, Perelman P, Driskell AC, Shapiro B, Xiong Z, Zeng Y, Liu S, Li Z, Liu B, Wu K, Xiao J, Yinqi X, Zheng Q, Zhang Y, Yang H, Wang J, Smeds L, Rheindt FE, Braun MJ, Fjeldså J, Orlando L, Barker FK, Jønsson KA, Johnson W, Koepfli K-P, O’Brien S, Haussler D, Ryder OA, Rahbek C, Willerslev E, Graves GR, Glenn TC, McCormack JE, Burt DW, Ellegren H, Alström P, Edwards SV, Stamatakis A, Mindell DP, Cracraft J, Braun EL, Warnow T, Jun W, Gilbert MTP, Zhang G (2014). Whole-genome analyses resolve early branches in the tree of life of modern birds. Science.

[6] Laumer CE, Hejnol A, Giribet G. Nuclear genomic signals of the ’microturbellarian’ roots of platyhelminth evolutionary innovation. eLife. 2015;4. https://doi.org/10.7554/eLife.05503. 10.7554/eLife.05503 PMC4398949 25764302

[7] Tarver JE, dos Reis M, Mirarab S, Moran RJ, Parker S, O’Reilly JE, King BL, O’Connell MJ, Asher RJ, Warnow T, Peterson KJ, Donoghue PCJ, Pisani D (2016). The Interrelationships of Placental Mammals and the Limits of Phylogenetic Inference. Genome Biol Evol.

[8] Rokas A, Williams BL, King N, Carroll SB (2003). Genome-scale approaches to resolving incongruence in molecular phylogenies. Nature.

[9] Springer MS, Gatesy J (2016). The gene tree delusion. Mol Phylogenet Evol.

[10] Meiklejohn KA, Faircloth BC, Glenn TC, Kimball RT, Braun EL (2016). Analysis of a Rapid Evolutionary Radiation Using Ultraconserved Elements: Evidence for a Bias in Some Multispecies Coalescent Methods. Syst Biol.

[11] Edwards SV, Xi Z, Janke A, Faircloth BC, McCormack JE, Glenn TC, Zhong B, Wu S, Lemmon EM, Lemmon AR, Leaché AD, Liu L, Davis CC (2016). Implementing and testing the multispecies coalescent model: A valuable paradigm for phylogenomics. Mol Phylogenet Evol.

[12] Shen X-X, Hittinger CT, Rokas A (2017). Contentious relationships in phylogenomic studies can be driven by a handful of genes. Nat Ecol Evol.

[13] Heled J, Drummond AJ (2010). Bayesian inference of species trees from multilocus data. Mol Biol Evol.

[14] Edwards SV (2009). Is a new and general theory of molecular systematics emerging?. Evolution.

[15] Pamilo P, Nei M (1988). Relationships between gene trees and species trees. Mol Biol Evol.

[16] Rannala B, Yang Z (2003). Bayes estimation of species divergence times and ancestral population sizes using DNA sequences from multiple loci. Genetics.

[17] Liu L, Yu L, Edwards SV (2010). A maximum pseudo-likelihood approach for estimating species trees under the coalescent model. BMC Evol Bioly.

[18] Liu L, Yu L (2011). Estimating species trees from unrooted gene trees. Syst Biol.

[19] Vachaspati P, Warnow T (2015). ASTRID: Accurate Species TRees from Internode Distances. BMC Genomics.

[20] Sayyari E, Mirarab S (2016). Anchoring quartet-based phylogenetic distances and applications to species tree reconstruction. BMC Genomics.

[21] Mirarab S, Reaz R, Bayzid MS, Zimmermann T, Swenson MS, Warnow T (2014). ASTRAL: genome-scale coalescent-based species tree estimation. Bioinformatics.

[22] Mirarab S, Warnow T (2015). ASTRAL-II: coalescent-based species tree estimation with many hundreds of taxa and thousands of genes. Bioinformatics.

[23] Liu L, Yu L, Pearl DK, Edwards SV (2009). Estimating species phylogenies using coalescence times among sequences. Syst Biol.

[24] Mossel E, Roch S (2010). Incomplete lineage sorting: consistent phylogeny estimation from multiple loci. IEEE/ACM Trans Comput Biol Bioinformatics (TCBB).

[25] Roch S, Warnow T (2015). On the robustness to gene tree estimation error (or lack thereof) of coalescent-based species tree methods. Syst Biol.

[26] Lafond M, Scornavacca C. On the Weighted Quartet Consensus problem. arXiv 610.00505. 2016.

[27] Allman ES, Degnan JH, Rhodes JA (2011). Determining species tree topologies from clade probabilities under the coalescent. J Theor Biol.

[28] Kane D, Tao T (2017). A Bound on Partitioning Clusters. Electr J Comb.

[29] Shekhar S, Roch S, Mirarab S (2017). Species tree estimation using ASTRAL: how many genes are enough?. IEEE/ACM Trans Comput Biol Bioinform.

[30] Davidson R, Vachaspati P, Mirarab S, Warnow T (2015). Phylogenomic species tree estimation in the presence of incomplete lineage sorting and horizontal gene transfer. BMC Genomics.

[31] Sayyari E, Mirarab S (2016). Fast Coalescent-Based Computation of Local Branch Support from Quartet Frequencies. Mol Biol Evol.

[32] Price MN, Dehal PS, Arkin AP (2010). FastTree-2 – Approximately Maximum-Likelihood Trees for Large Alignments. PLoS ONE.

[33] Mirarab S, Bayzid MS, Boussau B, Warnow T (2014). Statistical binning enables an accurate coalescent-based estimation of the avian tree. Science.

[34] Bayzid M. S, Mirarab S, Boussau B, Warnow T (2015). Weighted statistical binning: enabling statistically consistent genome-scale phylogenetic analyses. PLoS ONE.

[35] Mirarab S, Bayzid MS, Warnow T (2016). Evaluating Summary Methods for Multilocus Species Tree Estimation in the Presence of Incomplete Lineage Sorting. Syst Biol.

[36] Patel S (2013). Error in phylogenetic estimation for bushes in the tree of life. J Phylogenet Evol Biol.

[37] Gatesy J, Springer MS (2014). Phylogenetic analysis at deep timescales: unreliable gene trees, bypassed hidden support, and the coalescence/concatalescence conundrum. Mol Phylogenet Evol.

[38] Yu Y, Warnow T, Nakhleh L (2011). Algorithms for MDC-based multi-locus phylogeny inference: beyond rooted binary gene trees on single alleles. J Comput Biol.

[39] Hart PE, Nilsson NJ, Raphael B (1968). A formal basis for the heuristic determination of minimum cost paths. IEEE Trans Syst Sci Cybernet.

[40] Zhang C, Sayyari E, Mirarab S, Meidanis J, Nakhleh L (2017). ASTRAL-III: Increased Scalability and Impacts of Contracting Low Support Branches. Lecture Notes in Computer Science. vol. 10562 LNBI.

[41] Stamatakis A (2014). RAxML version 8: A tool for phylogenetic analysis and post-analysis of large phylogenies. Bioinformatics.

[42] Mallo D, De Oliveira Martins L, Posada D (2016). SimPhy : Phylogenomic Simulation of Gene, Locus, and Species Trees. Syst Biol.

[43] Fletcher W, Yang Z (2009). INDELible: A flexible simulator of biological sequence evolution. Mol Biol Evol.

[44] Tavaré S (1986). Some probabilistic and statistical problems in the analysis of DNA sequences. Lect Math Life Sci.

[45] Junier T, Zdobnov EM (2010). The Newick utilities: high-throughput phylogenetic tree processing in the UNIX shell. Bioinformatics.

[46] Robinson D, Foulds L (1981). Comparison of phylogenetic trees. Math Biosci.

[47] Kozlov AM, Aberer AJ, Stamatakis A (2015). ExaML version 3: a tool for phylogenomic analyses on supercomputers. Bioinformatics.

