# Supplementary material for: ASTRAL-III: polynomial time species tree reconstruction from partially resolved gene trees
Source: BMC Bioinformatics. 2018 May 8;19(Suppl 6):153. doi: 10.1186/s12859-018-2129-y (PMC5998893; doi:10.1186/s12859-018-2129-y)
Supplement: Supplementary file 1 — Supplementary material and appendices. Appendices A, B, and C in addition to Figures S1–S8, and Tables S1–S4 are all provided as one Additional file 1. (PDF 476 kb) [file 12859_2018_2129_MOESM1_ESM.pdf]

SUPPLEMENTARY MATERIAL FOR "ASTRAL-III: POLYNOMIAL TIME SPECIES TREE RECONSTRUCTION FROM PARTIALLY RESOLVED GENE TREES"

CHAO ZHANG AND MARYAM RABIEE AND ERFAN SAYYARI AND SIAVASH MIRARAB

Contents

Appendix A. Supplementary method details 2
A.1. Defining the set X 2
A.2. Similarity matrix 2
A.3. Greedy trees 2
A.4. Gene tree polytomies 2
Appendix B. Derivations 4
B.1. Derivation of Equation 6 4
B.2. Derivation of the upperbound $U(Z)$ 4
Appendix C. Simulations and commands 7
C.1. Simulation setup 7
C.1.1. S100 7
C.1.2. Larege-n simulated dataset 7
C.2. Commands 8
C.2.1. Contracting branches 8
C.2.2. Drawing bootstrap support on ML gene trees: 8
C.2.3. Gene tree estimation 8
C.2.4. Running ASTRAL 9
Appendix D. Supplementary Figures and Tables 10

Appendix A. Supplementary method details

A.1. **Defining the set X.** ASTRAL-II uses several techniques to augment the set X, which we describe below. We also describe how ASTRAL-III modifies each technique.

A.2. **Similarity matrix.** All bipartitions from a UPGMA tree based on a quartet-based measure of distance are added to X. In ASTRAL-III, we improve the distance matrix when gene trees have polytomies. Unlike ASTRAL-II, in ASTRAL-III we make sure that unresolved quartets in input gene trees contribute exactly 0 to our counts of different quartet topologies used in building the similarity matrix. Note that this similarity matrix is separate from and has no impact on the quartet scores.

A.3. **Greedy trees.** ASTRAL-II uses a set of heuristics based on the greedy consensus of gene trees to augment the set X. It first constructs a set of greedy consensus trees using a set of thresholds for minimum frequency of bipartitions. The polytomies in the greedy consensus trees are resolved in three different ways and resulting bipartitions are added to X (see Algorithm S1). Of the methods used to resolve the polytomy with degree d, two of them (i.e., using a UPGMA tree started from sides of the polytomy and a greedy consensus of gene trees subsampled to randomly selected taxa) can only add $O(d)$ new bipartitions. The third resolution samples a taxon from each side of the polytomy; it then computes a caterpillar tree constructed based on decreasing similarity to each sampled taxon and adds the bipartitions from all these caterpillar trees to the search space. This step can add $O(d^2)$ bipartitions to the search space. In ASTRAL-III, to guarantee $|X| = O(nk)$, we need to constrain this step. Let $d_1 \ldots d_r$ be the list of all polytomies, ordered from the smallest to the largest. Then, we find the smallest threshold q such that $\sum_{i=1}^{q} d_i^2 \leq cn$ for a constant c, set by default to 25. In ASTRAL-III, we only compute and add bipartitions using caterpillar resolutions for polytomies $d_1 \ldots d_q$ (see Algorithm S1). By construction, this will ensure that at most $O(n)$ bipartitions are added in this step. Finally, these resolutions can happen in multiple rounds. In ASTRAL-III, we make sure these rounds of resolutions do not grow beyond a constant (default: 100).

A.4. **Gene tree polytomies.** If a gene tree includes polytomies, ASTRAL-II adds bipartitions implied by resolutions of that polytomy to the set X. ASTRAL-II computes a single "reference" tree by computing a greedy consensus of all gene trees and forcing the consensus to be fully resolved with further refinements using the UPGMA algorithm. To resolve a gene tree polytomy, it samples a taxon from each cluster defined by each side of the polytomy, finds the reference tree induced on the sampled taxa, and adds the resulting resolution to the search space. In ASTRAL-III, the definition of the reference tree is modified to use the UPGMA tree inferred on the similarity matrix used by ASTRAL. We observed that the UPGMA tree summarizes the input gene trees more accurately than the greedy trees (Table S1). Moreover, unlike ASTRAL-II, in ASTRAL-III, this process is repeated three times with different random samplings.

Algorithm S1 - Additions to X using greedy consensus. $greedy(\mathcal{G}, t, b)$ returns the greedy consensus of $\mathcal{G}$, including only branches with frequency $\geq t$; if b is true, polytomies in the consensus are randomly resolved. $updateX(t)$ adds bipartitions from tree t to the set X; when edges in t are labelled with a frequency label (e.g., frequencies in the greedy consensus), it returns the maximum label of any *new* bipartition added to X. $clusters(p)$ returns the taxon partitions defined by an unrooted node p. $upgma(S, C)$ runs the UPGMA algorithm using the similarity matrix S; when C is given, UPGMA starts by groups defined in C. $randSample(p)$ selects a random taxon from each subtree around a node p, and $resolve(p, r)$ resolves polytomy p according to a tree r on such a sampling. Operator $\upharpoonright$ restricts a tree or a matrix to a subset. $pectinate(O)$ returns a pectinate tree based on O, an ordered list of taxa. $sortBy$ sorts a list of taxa based on their decreasing similarity to a given taxon. Constants: $THS = \{0, \frac{1}{100}, \frac{1}{50}, \frac{1}{20}, \frac{1}{10}, \frac{1}{4}, \frac{1}{3}\}$; $MIT = 10$; $RWD = 2$; and $FRQ = LTH = \frac{1}{100}$; $MAXR = 100$.

```
function addByGreedy(𝒢, S)
    for t ∈ THS do
        gc ← greedy(𝒢, t, False)
        for p ∈ polytomies(gc) do
            if degree(p) ≥ POLYLIMIT then
                quadratic ← FALSE
            else
                quadratic ← True
            updateX(upgma(S, start = clusters(p)))
            c ← 0 and max ← MIT
            while c < max do
                c ← c + 1
                sample ← randSample(p)
                r ← greedy(𝒢 ↾ sample, 0, True)
                mt ← updateX(resolve(p, r))
                if mt ≥ FRQ AND max ≤ MAXR then
                    max ← max + RWD
                updateX(resolve(p, upgma(S ↾ sample)))
                if t ≤ LTH and c < MIT and quadratic then
                    for s ∈ sample do
                        r ← pectinate(sortBy(S, s, sample)
                        updateX(resolve(p, r))
```

Appendix B. Derivations

B.1. **Derivation of Equation 6.** First note that:

$$
\begin{aligned}
QI((A|B|C),M) =&\sum_{i\in[d]}\sum_{j\in[d]-\{i\}}\sum_{k\in[d]-\{i,j\}}\frac{a_i+b_j+c_k-3}{2}a_ib_jc_k\\
=&\sum_{i\in[d]}\binom{a_i}{2}\sum_{j\in[d]-\{i\}}\sum_{k\in[d]-\{i,j\}}b_jc_k\\
&+\sum_{i\in[d]}\binom{b_i}{2}\sum_{j\in[d]-\{i\}}\sum_{k\in[d]-\{i,j\}}a_jc_k\\
&+\sum_{i\in[d]}\binom{c_i}{2}\sum_{j\in[d]-\{i\}}\sum_{k\in[d]-\{i,j\}}a_jb_k\ .
\end{aligned}
\tag{1}
$$

Now, we note that:

$$
\begin{aligned}
&\sum_{j\in[d]-\{i\}}\sum_{k\in[d]-\{i,j\}}b_jc_k\\
=&\sum_{j\in[d]-\{i\}}b_j\sum_{k\in[d]-\{i,j\}}c_k\\
=&\sum_{j\in[d]-\{i\}}b_j(S_c-c_i-c_j)\\
=&-b_i(S_c-c_i-c_i)+\sum_{j\in[d]}b_j(S_c-c_i-c_j)\\
=&2b_ic_i-S_cb_i+S_bS_c-S_bc_i-S_{b,c}\\
=&(S_b-b_i)(S_c-c_i)-S_{b,c}+b_ic_i
\end{aligned}
\tag{2}
$$

Replacing this (ditto for other terms) in Equation 1 directly gives us the Equation 6:

$$
\begin{aligned}
QI((A|B|C),M) =&\sum_{i\in[d]}\binom{a_i}{2}((S_b-b_i)(S_c-c_i)-S_{b,c}+b_ic_i)\\
&+\sum_{i\in[d]}\binom{b_i}{2}((S_a-a_i)(S_c-c_i)-S_{a,c}+a_ic_i)\\
&+\sum_{i\in[d]}\binom{c_i}{2}((S_a-a_i)(S_b-b_i)-S_{a,b}+a_ib_i)
\end{aligned}
\tag{3}
$$

B.2. **Derivation of the upperbound $U(Z)$.** In ASTRAL, $V(Z)$ denotes the total contribution to the support of the best rooted tree T_Z on taxon set Z, where each quartet tree in the set of input gene trees contributes 0 if it conflicts with T_Z or only intersects it with one leaf, and otherwise contributes 1 or 2, depending on the number of nodes in

T_Z it maps to. Let $U(Z)$ be the sum of max possible support of each quartet tree in the gene trees with respect to any resolution T_Z of set Z, allowing the resolution to change for each gene tree. In other words, let $Q(Z)$ be the set of quartets that would be resolved one way or another in any resolution of Z, and note that these are quartets that include two or leaves in Z; then, $U(Z)$ is the number of resolved gene tree quartets that would match *some* resolution of Z and are included in $Q(Z)$. More formally,

$$U(Z) = \sum_{g\in G} \sum_{M\in N(g)} \sum_{T\in Q(Z)} QI(T, M) \ ,$$

where

$$\begin{aligned} Q_1(Z) &= \{\{\{v,w\},\{x\},\{y\}\} : \{x,y\} \subset Z, \{v,w\} \subset L - \{x,y\}\} \ , \\ Q_2(Z) &= \{\{\{v,w\},\{x\},\{y\}\} : \{v,w,x\} \subset Z, y \in L - Z\} \ , \text{ and} \\ Q(Z) &= Q_1(Z) \cup Q_2(Z) \ , Q_1(Z) \cap Q_2(Z) = \emptyset \ . \end{aligned}$$

Clearly, $V(Z) \leq U(Z)$ (equality can be achieved only if all gene trees are compatible with some resolution of Z). Then, letting $d = |M|$ and defining $z_i = |Z \cap M_i|$ and $l_i = |L \cap M_i| = |M_i|$, we have:

$$\begin{aligned}
&\sum_{\{A,B,C\}\in Q(Z)} QI((A|B|C), M) \\
=&\sum_{\{A,B,C\}\in Q_1(Z)} QI((A|B|C), M) + \sum_{\{A,B,C\}\in Q_2(Z)} QI((A|B|C), M) \\
=&\sum_{i\in[d]} \sum_{j\in[d]-\{i\}} \sum_{k\in[d]-\{i\}-[j]} \binom{l_i}{2} z_j z_k \\
&+\sum_{i\in[d]} \sum_{j\in[d]-\{i\}} \sum_{k\in[d]-\{i\}-[j]} \binom{z_i}{2} (z_j(l_k - z_k) + (l_j - z_j)z_k) \\
=&\sum_{i\in[d]} \sum_{j\in[d]-\{i\}} \sum_{k\in[d]-\{i,j\}} \binom{l_i}{2} \frac{z_j z_k}{2} \\
&+\sum_{i\in[d]} \sum_{j\in[d]-\{i\}} \sum_{k\in[d]-\{i,j\}} \binom{z_i}{2} \frac{z_j(l_k - z_k) + (l_j - z_j)z_k}{2} \\
=&\sum_{i\in[d]} \sum_{j\in[d]-\{i\}} \sum_{k\in[d]-\{i,j\}} \binom{l_i}{2} \frac{z_j z_k}{2} \\
&+\sum_{i\in[d]} \sum_{j\in[d]-\{i\}} \sum_{k\in[d]-\{i,j\}} \binom{z_i}{2} z_j(l_k - z_k) \ .
\end{aligned} \tag{4}$$

Notice that based on Equation 4,

$$
\begin{aligned}
&\frac{QI((Z|Z|L),M)}{2} - \frac{QI((Z|Z|Z),M)}{3} = \\
&\frac{1}{2}\sum_{i\in[d]}\sum_{j\in[d]-\{i\}}\sum_{k\in[d]-\{i,j\}} z_i z_j l_k \frac{z_i+z_j+l_k-3}{2} = \\
&-\frac{1}{3}\sum_{i\in[d]}\sum_{j\in[d]-\{i\}}\sum_{k\in[d]-\{i,j\}} z_i z_j z_k \frac{z_i+z_j+z_k-3}{2} = \\
&\frac{1}{2}\sum_{i\in[d]}\sum_{j\in[d]-\{i\}}\sum_{k\in[d]-\{i,j\}} (\binom{z_i}{2} z_j l_k + z_i \binom{z_j}{2} l_k + z_i z_j \binom{l_k}{2}) \\
&-\frac{1}{3}\sum_{i\in[d]}\sum_{j\in[d]-\{i\}}\sum_{k\in[d]-\{i,j\}} (\binom{z_i}{2} z_j z_k + z_i \binom{z_j}{2} z_k + z_i z_j \binom{z_k}{2}) = \\
&\frac{1}{2}\sum_{i\in[d]}\sum_{j\in[d]-\{i\}}\sum_{k\in[d]-\{i,j\}} (\binom{z_i}{2} z_j l_k + \binom{z_i}{2} z_j l_k + \binom{l_i}{2} z_j z_k) \\
&-\frac{1}{3}\sum_{i\in[d]}\sum_{j\in[d]-\{i\}}\sum_{k\in[d]-\{i,j\}} (\binom{z_i}{2} z_j z_k + \binom{z_i}{2} z_j z_k + \binom{z_i}{2} z_j z_k) = \\
&\frac{1}{2}\sum_{i\in[d]}\sum_{j\in[d]-\{i\}}\sum_{k\in[d]-\{i,j\}} (\binom{l_i}{2} z_j z_k + 2\binom{z_i}{2} z_j l_k) \\
&-\frac{1}{3}\sum_{i\in[d]}\sum_{j\in[d]-\{i\}}\sum_{k\in[d]-\{i,j\}} 3\binom{z_i}{2} z_j z_k = \\
&\sum_{A,B,C\in Q(Z)} QI((A|B|C),M)\ .
\end{aligned} \tag{5}
$$

(going from the fourth term to the fifth is accomplished by changing the order of sums).

Therefore,

$$
\begin{aligned}
U(Z) &= \sum_{g\in G}\sum_{M\in N(g)} (\frac{QI((Z|Z|L),M)}{2} - \frac{QI((Z|Z|Z),M)}{3}) \\
&= \frac{w(Z|Z|L)}{2} - \frac{w(Z|Z|Z)}{3}\ .
\end{aligned} \tag{6}
$$

Appendix C. Simulations and commands

C.1. Simulation setup.

C.1.1. *S100.* In order to generate the gene trees and species trees using the Simphy we use this command:

```
simphy -rs 50 -rl f:1000 -rg 1 -sb f:0.0000001 -sd f:0
-st ln:14.70055,0.25 -sl f:100 -so f:1 -si f:1 -sp
f:400000 -su ln:-17.27461,0.6931472 -hh f:1 -hs ln:1.5,1
-hl ln:1.551533,0.6931472 -hg ln:1.5,1 -cs 9644 -v 3
-o ASTRALIII -ot 0 -op 1 -od 1
```

C.1.2. *Larege-n simulated dataset.* In order to compare running time performances of ASTRAL-II and ASTRAL-III, we created another dataset with very large numbers of species using Simphy and under the MSCM. Since we are only comparing running times, we only use true gene trees to infer the ASTRAL species trees. We have three sub-datasets with 5000, 2000, and 1000 species (plus one outgroup). Each sub-dataset has 4 replicates, and each replicate has a different species tree with 500 gene trees. Species trees are generated based on the birth-death process with birth and date rates from log uniform distributions. We sampled the number of generations and effective population size from log normal and uniform distributions respectively such that we have medium amounts of ILS. The average FN rates between the true gene trees and the species tree ranges between 4% and 23% for 1K, between 21% and 58% for 2k, and between 21% and 33% for 5k.

In order to generate the gene trees and true species trees using the Simphy we use parameters given in Table S4 and the following command.

1K:

```
simphy -rs 20 -rl f:1000 -rg 1 -sb lu:0.0000001,0.000001 -sd
lu:0.0000001,sb -st ln:16,1 -sl f:1000 -so f:1 -si f:1 -sp
u:10000,1000000 -su ln:-17.27461,0.6931472 -hh f:1 -hs ln:1.5,1 -hl
ln:1.551533,0.6931472 -hg ln:1.5,1 -cs 9644 -v 3 -o 5k.species -ot 0
-op 1 -od 1
```

2K:

```
simphy -rs 20 -rl f:1000 -rg 1 -sb lu:0.0000001,0.000001 -sd
lu:0.0000001,sb -st ln:16,1 -sl f:2000 -so f:1 -si f:1 -sp
u:10000,1000000 -su ln:-17.27461,0.6931472 -hh f:1 -hs ln:1.5,1 -hl
ln:1.551533,0.6931472 -hg ln:1.5,1 -cs 9644 -v 3 -o 5k.species -ot 0
-op 1 -od 1
```

5K:

```
simphy −rs 20 −rl f:1000 −rg 1 −sb lu:0.0000001,0.000001 −sd
lu:0.0000001,sb −st ln:16,1 −sl f:5000 −so f:1 −si f:1 −sp
u:10000,1000000 −su ln:−17.27461,0.6931472 −hh f:1 −hs ln:1.5,1 −hl
ln:1.551533,0.6931472 −hg ln:1.5,1 −cs 9644 −v 3 −o 5k.species −ot 0
−op 1 −od 1
```

10K: For the 10K-taxon dataset of S2 we use this command

```
simphy −rs 20 −rl f:1000 −rg 1 −sb lu:0.0000001,0.000001 −sd
lu:0.0000001,sb −st ln:16.2,1 −sl f:10000 −so f:1 −si f:1 −sp
u:10000,1000000 −su ln:−17.27461,0.6931472 −hh f:1 −hs ln:1.5,1 −hl
ln:1.551533,0.6931472 −hg ln:1.5,1 −cs 9644 −v 3 −o 10k.species −ot
0 −op 1 −od 1
```

C.2. **Commands.**

C.2.1. *Contracting branches.* In order to contract gene tree branches with bootstrap up to a certain threshold we used this command:

```
nw_ed genetree 'i & (b<=$threshold)' o
```

C.2.2. *Drawing bootstrap support on ML gene trees:* In order to draw bootstrap support on best ML gene trees we first reroot both best ML gene tree, and the bootstrap gene trees using this command:

```
nw_support bootstrapgenetrees taxon > bootstrapgenetrees.rerooted
nw_support bestMLgenetree taxon > bestMLgenetree.rerooted
```

Then we draw bootstrap supports on the branches:

```
nw_support −p bestMLgenetree.rerooted bootstrapgenetrees.rerooted >
bestMLgenetree.rerooted.final
```

C.2.3. *Gene tree estimation.* We used FastTree version 2.1.9 Double precision. In order to estimated best ML gene trees we used the following command:

```
fasttree −nt −gtr −nopr −gamma −n <num> <all−genes.phylip>
```

where we have all the alignments in the PHYLIP format in the file all-genes.phyip for each replicate, and $< num >$ is the number of alignments in this file.

For bootstrapping analysis, we first generate bootstrapped sequences using RAxML version 8.2.9 with the following command:

```
raxmlHPC −s alignment.phylip −f j
         −b <seed number> −n BS −m GTRGAMMA −# 100
```

and then we Fasttree to perform the actual ML analyses; for FastTree bootstrap runs, we use the same command and models that we used for best ML gene trees.

C.2.4. *Running ASTRAL.* ASTRAL-II in this paper refers to ASTRAL version 4.11.2 and ASTRAL-III refers to ASTRAL version 5.5.4. Both versions can be found in the link below:

```
https://github.com/chaoszhang/ASTRAL/releases/tag/paper
```

Both versions of ASTRAL program were run with following command:

```
java -jar <program> -t 0 -i <input> -o <output> &> <log>
```

Appendix D. Supplementary Figures and Tables

Table S1. The accuracy of UPGMA tree and Greedy tree of two model conditions of dataset S100

Contraction threshold	Greedy tree RF	UPGMA tree RF
0%	0.168	0.1461
10%	0.169	0.1451

Table S2. Species tree and gene tree generation parameters used for Simphy, and sequence evolution parameters for the GTR model used for Indelible for the S100 dataset.

Parameter Name	parameter Value
Speciation rate	0.0000001
Extinsion rate	0
Number of Leaves	100
Ingroup divergence to the ingroup ratio	1.0
Generations	LogN(1.470055e+01,2.500000e-01)
Haploid effective population size	400000
Global substitution rate	LogN(-1.727461e+01,6.931472e-01)
Lineage specific rate gamma shape	LogN(1.500000e+00,1)
Gene family specific rate gamma shape	LogN(1.551533e+00,6.931472e-01)
Gene tree branch specific rate gamma shape	LogN(1.500000e+00,1)
Seed	9644
Sequence Length	1600, 800, 400, 200
Sequence base frequencies	Dirichlet(A=36,C=26,G=28,T=32)
Sequence transition rates	Dirichlet(TC=16,TA=3,TG=5,CA=5,CG=6,AG=15)

TABLE S3. Species tree error (FN ratio) for all model conditions of the S100 dataset, with true gene trees (*true*), no filtering (*non*), and all filtering thresholds (*columns*).

Genes	Alignment	true	non	0	3	5	7	10	20	33	50	75
50	200bp	7.0	17.4	**15.7**	16.1	16.0	16.1	16.8	16.9	19.0	22.9	31.4
50	400bp		13.4	13.2	**12.8**	**12.8**	13.0	**12.8**	13.6	14.3	16.4	20.7
50	800bp		12.0	11.7	11.3	11.1	11.0	11.0	**10.9**	11.7	12.4	15.4
50	1600bp		10.2	10.2	10.1	10.1	**9.8**	9.9	10.0	10.0	10.4	11.9
200	200bp	3.7	11.3	10.4	**10.1**	10.3	10.3	10.4	10.3	12.1	14.3	20.5
200	400bp		9.0	8.3	8.3	**8.0**	**8.0**	8.2	8.3	8.8	9.8	12.9
200	800bp		7.4	7.2	6.9	6.9	6.9	**6.8**	6.9	7.2	7.5	8.9
200	1600bp		6.1	6.3	6.2	6.2	6.1	6.1	**5.9**	6.1	6.2	7.3
500	200bp	2.4	9.5	8.5	8.4	8.5	**8.1**	**8.1**	**8.1**	9.4	10.9	15.7
500	400bp		7.1	6.7	6.4	**6.3**	6.5	**6.3**	6.5	6.7	7.7	9.9
500	800bp		5.7	5.4	5.3	5.4	5.2	5.3	**5.1**	**5.1**	5.6	6.4
500	1600bp		4.8	4.6	4.5	4.5	**4.3**	4.4	4.4	**4.3**	4.5	5.0
1000	200bp	1.5	8.8	7.8	7.2	7.2	7.0	**6.9**	7.1	7.9	9.2	12.5
1000	400bp		6.7	5.9	5.5	5.6	5.3	5.4	**5.2**	5.4	6.3	7.9
1000	800bp		5.3	4.9	4.8	4.7	4.8	4.7	**4.3**	4.4	4.5	5.4
1000	1600bp		4.1	4.2	4.0	3.8	3.9	**3.7**	**3.7**	3.8	3.8	4.1

TABLE S4. Species tree and gene tree generation parameters in Simphy for 1K-taxon, 2K-taxon and 5K-taxon datasets

Parameter Name	parameter Value
Speciation rate	LogU[1.000000e-07,1.000000e-06)
Extinsion rate	LogU[1.000000e-07,SB)
Locus trees	1000
Gene trees	1
Number of Leaves	1000, 2000, or 5000
Ingroup divergence to the ingroup ratio	1.0
Generations	LogN(16,1)
Haploid effective population size	Uniform[10000,1000000]
Global substitution rate	LogN(-1.727461e+01,6.931472e-01)
Lineage specific rate gamma shape	LogN(1.500000e+00,1)
Gene family specific rate gamma shape	LogN(1.551533e+00,6.931472e-01)
Gene tree branch specific rate gamma shape	LogN(1.500000e+00,1)
Seed	9644

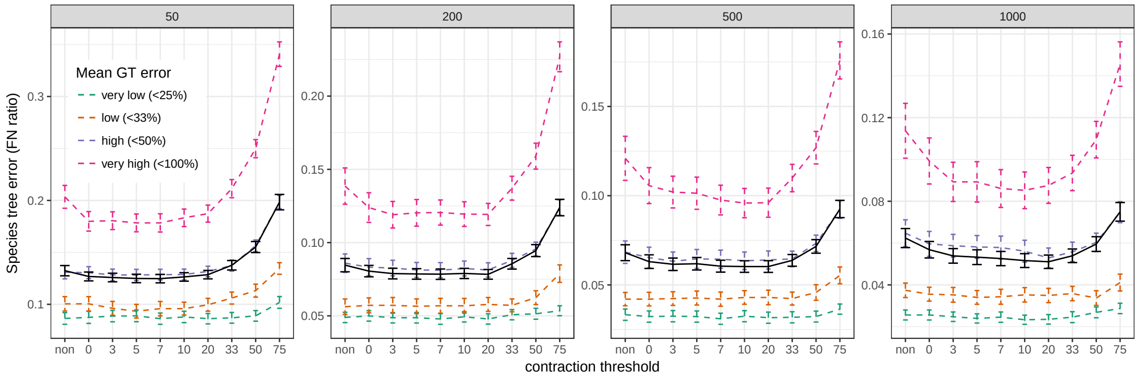

Figure S1. Impact of contraction on the S100 dataset. The error in species trees estimated by ASTRAL-III on the S100 dataset given $k = 50$, 200, 500, or 1000 genes (*boxes*) and with full FastTree gene trees (*non*) or trees with branches with $\leq \{0, 3, 5, 7, 10, 20, 33, 50\}\%$ support contracted (*x-axis*). Average FN error and standard error bars are shown for all 50 replicates with the four alignment lengths combined (*black solid line*); average FN error broken down by gene tree error is also shown (*dashed colored lines*). We divide the replicates based on their average gene tree error (normalized RF) into four categories: $[0, \frac{1}{4}]$,$(\frac{1}{4}, \frac{1}{3}]$,$(\frac{1}{3}, \frac{1}{2}]$,$(\frac{1}{2}, 1]$.

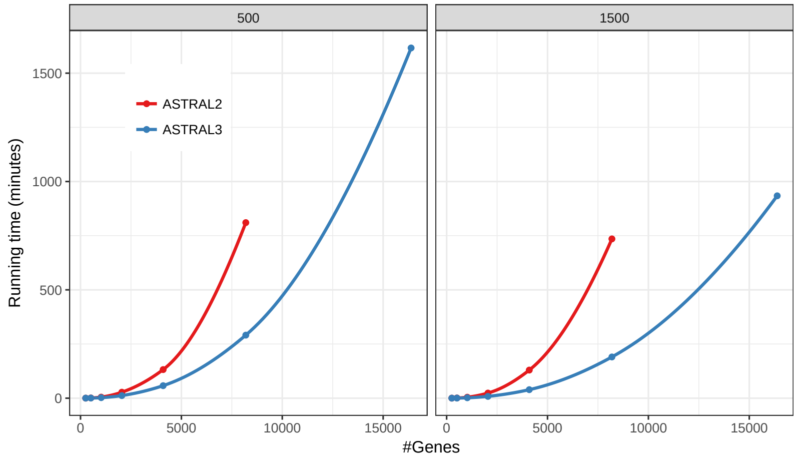

FIGURE S2. Running time versus k. Average running time of ASTRAL-II versus ASTRAL-III on the avian dataset with 500bp or 1500bp alignments with varying numbers of gens (k), shown in normal scale. A LOESS curve is fit to the data points. ASTRAL-II could not finish on 2^{14} genes in the allotted 48-hour time slot. Averages are over 4 runs.

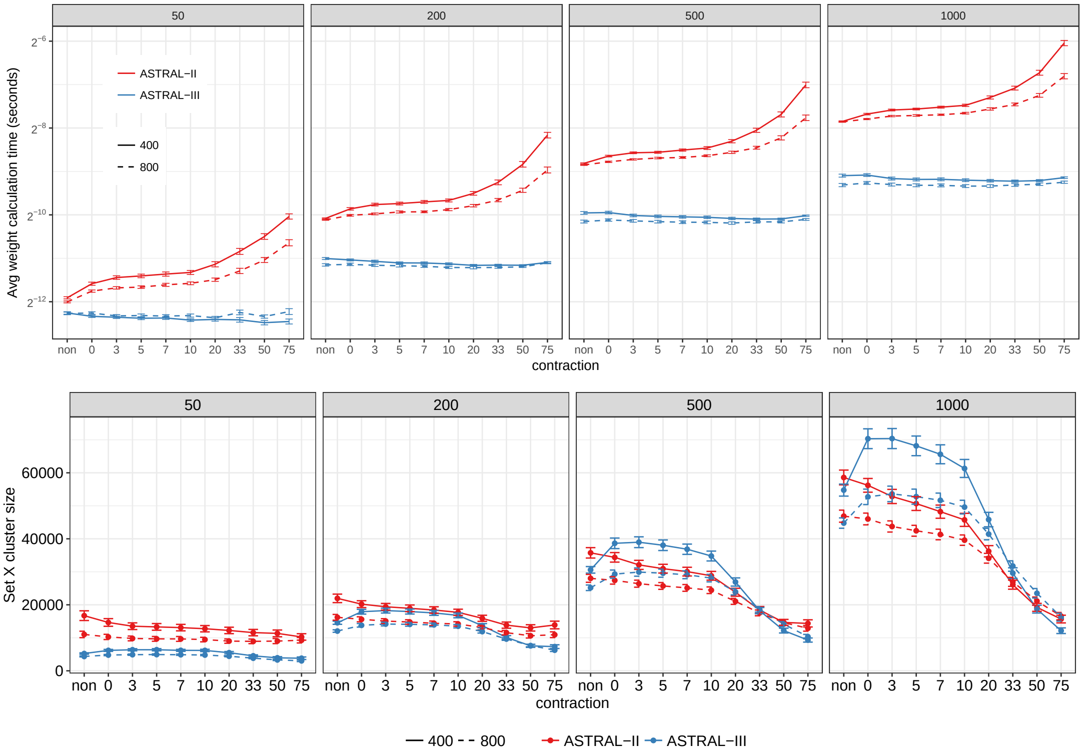

(a)

(b)

Figure S3. Weight calculation and $|X|$ on S100. Average and standard error of (a) the time it takes to score a single tripartition using Eq. 3 and (b) search space size $|X|$ for both ASTRAL-II (red) and ASTRAL-III (blue) on the S100 dataset. Running time is in log scale for varying numbers of gene trees (*boxes*) and sequence length 400 and 800 (*line types*).

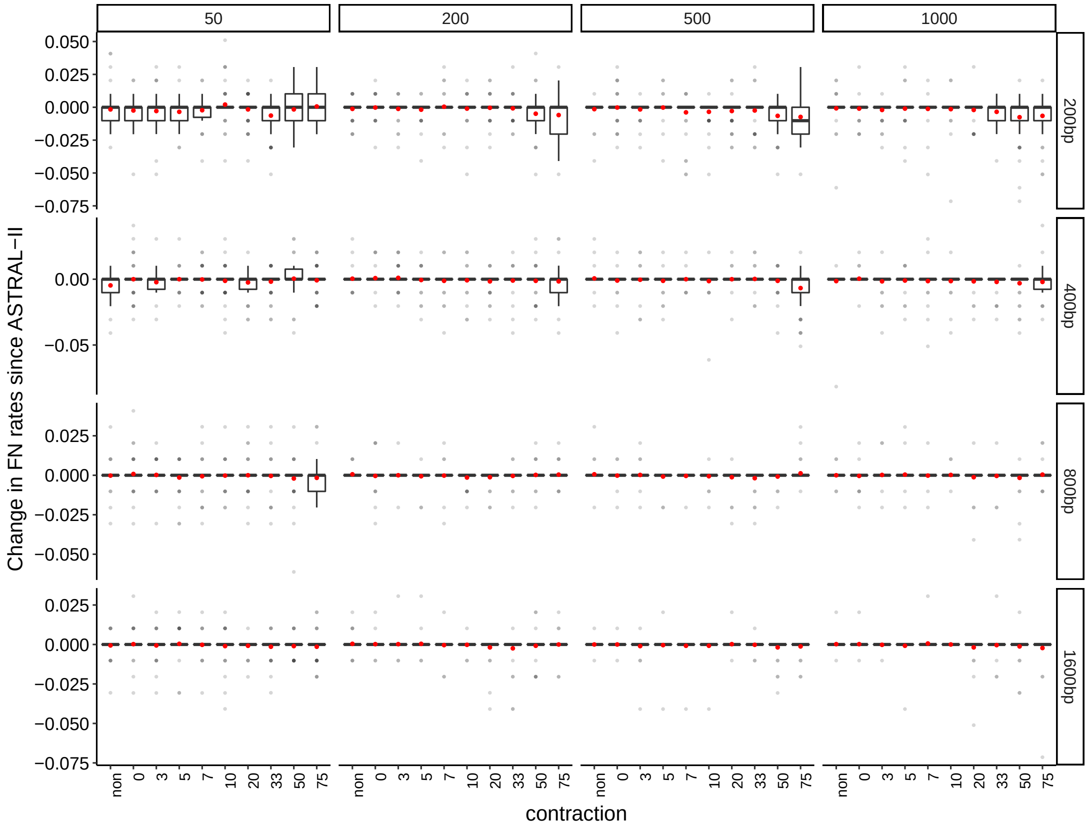

FIGURE S4. Change in species tree FN rates between ASTRAL-II and ASTRAL-III (ASTRAL-III-ASTRAL-II) for S100 dataset varying number of genes, number of base pairs, and contraction levels. Negative values indicate improvements over ASTRAL-II.

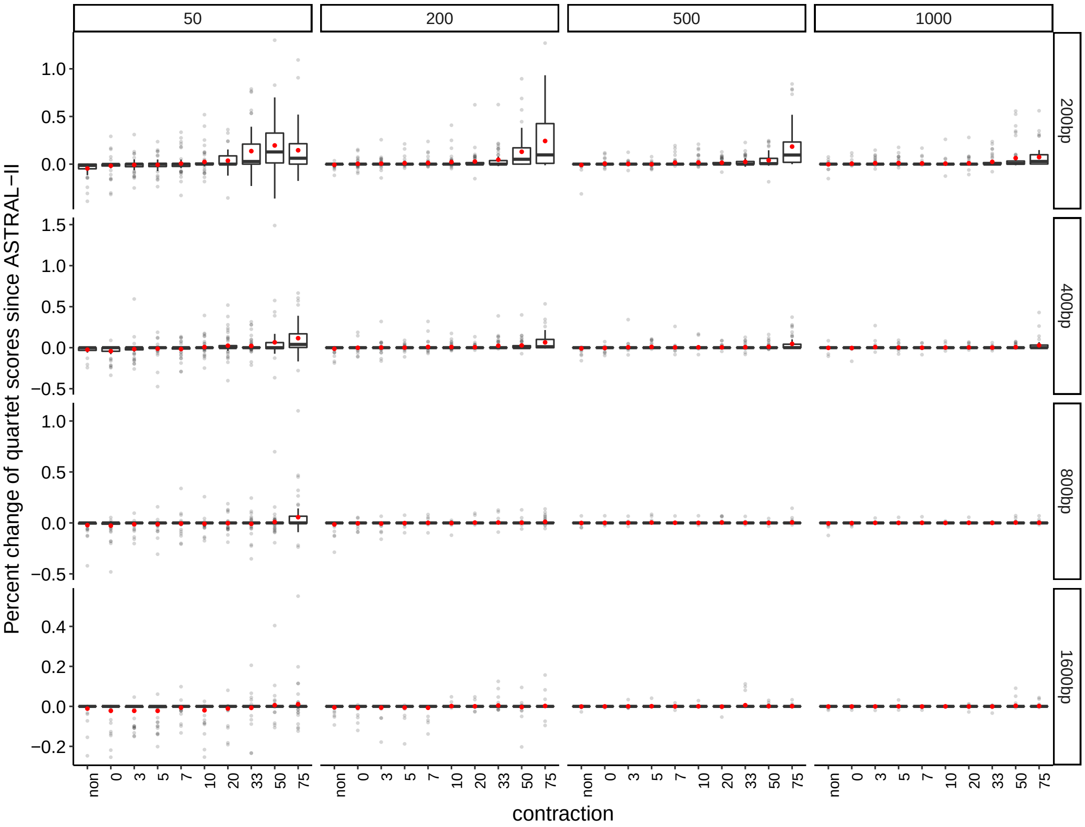

Figure S5. Percent change in species tree quartet scores between ASTRAL-II and ASTRAL-III ($\frac{\text{ASTRAL-III-ASTRAL-II}}{\text{ASTRAL-II}} \times 100$) for S100 dataset varying number of genes, number of base pairs, and contraction levels. Positive values indicate improvements over ASTRAL-II.

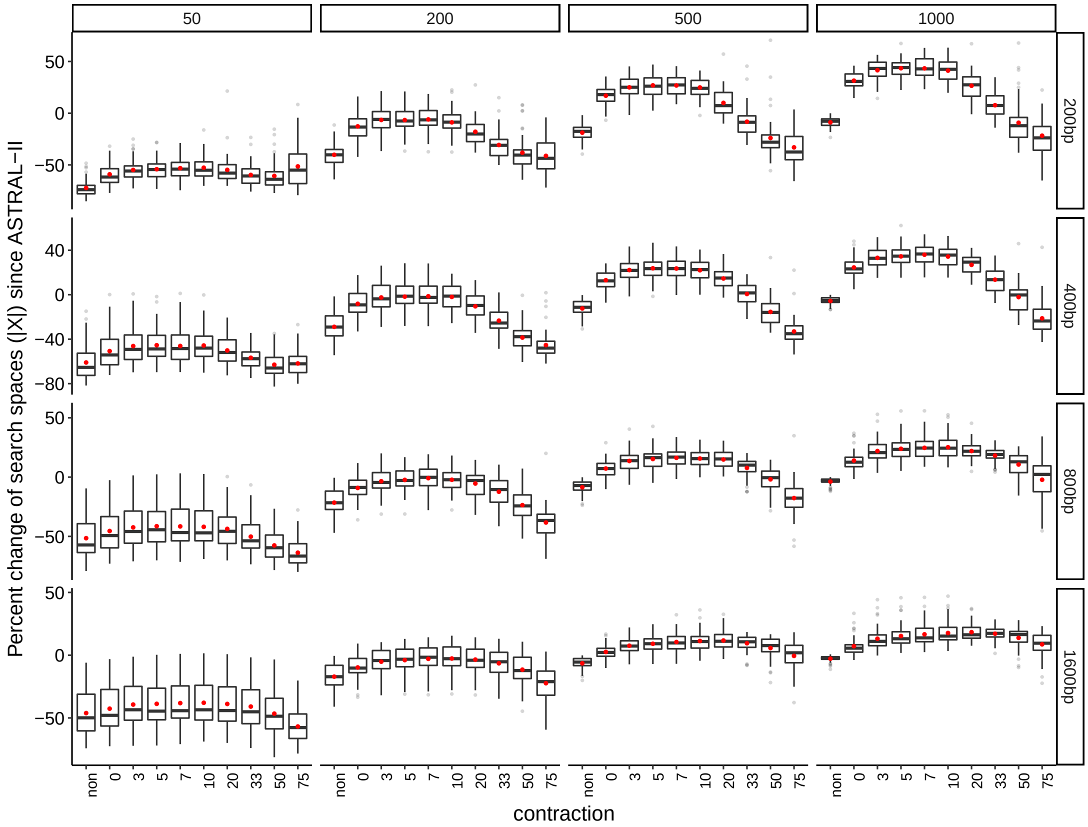

FIGURE S6. Percent change in species tree search space ($|X|$) between ASTRAL-II and ASTRAL-III ($\frac{\text{ASTRAL-III-ASTRAL-II}}{\text{ASTRAL-II}} \times 100$) for S100 dataset varying number of genes, number of base pairs, and contraction levels. Positive values indicate larger search space over ASTRAL-II.

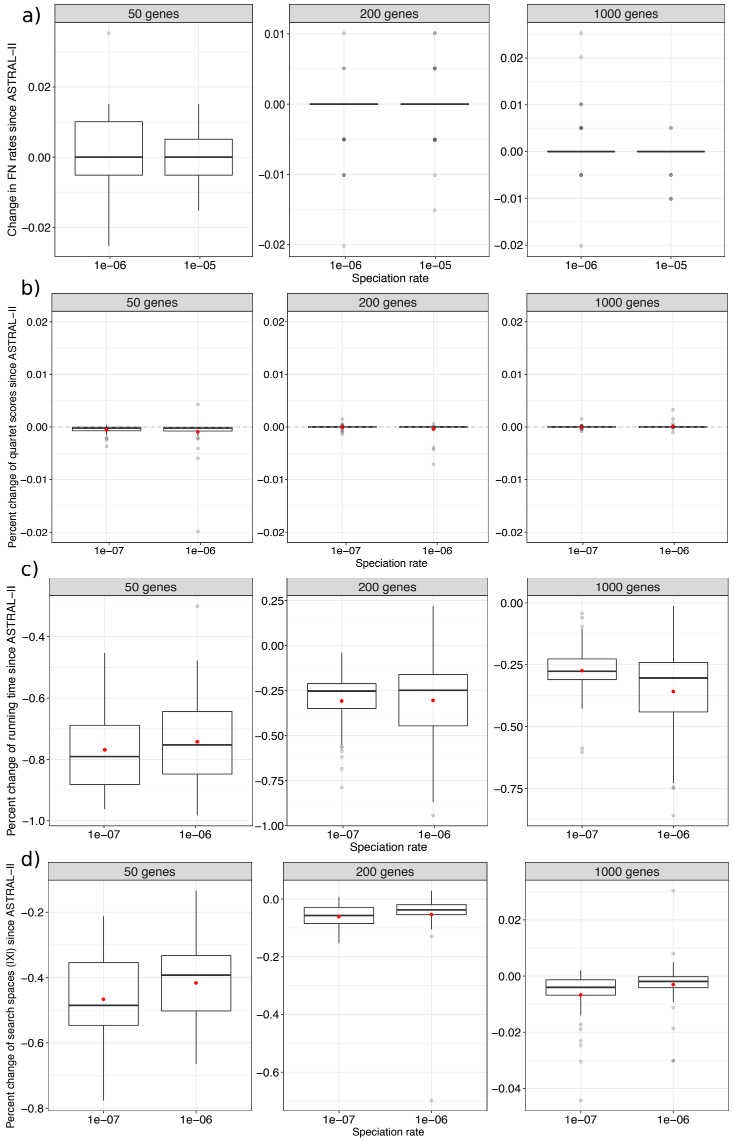

Figure S7. (a) Change in species tree FN rates between ASTRAL-II and ASTRAL-III (ASTRAL-III-ASTRAL-II) for S200 dataset. Negative values indicate improvements over ASTRAL-II. (b) Percent change in species tree quartet scores between ASTRAL-II and ASTRAL-III ($\frac{\text{ASTRAL-III-ASTRAL-II}}{\text{ASTRAL-II}} \times 100$) for S200 dataset. Positive values indicate improvements over ASTRAL-II. (c) Percent change in running time between ASTRAL-II and ASTRAL-III ($\frac{\text{ASTRAL-III-ASTRAL-II}}{\text{ASTRAL-II}} \times 100$) for S200 dataset. Positive values indicate longer running times over ASTRAL-II. (d) Percent change in species tree search space ($|X|$) between ASTRAL-II and ASTRAL-III ($\frac{\text{ASTRAL-III-ASTRAL-II}}{\text{ASTRAL-II}} \times 100$) for S200 dataset. Positive values indicate larger search space over ASTRAL-II.

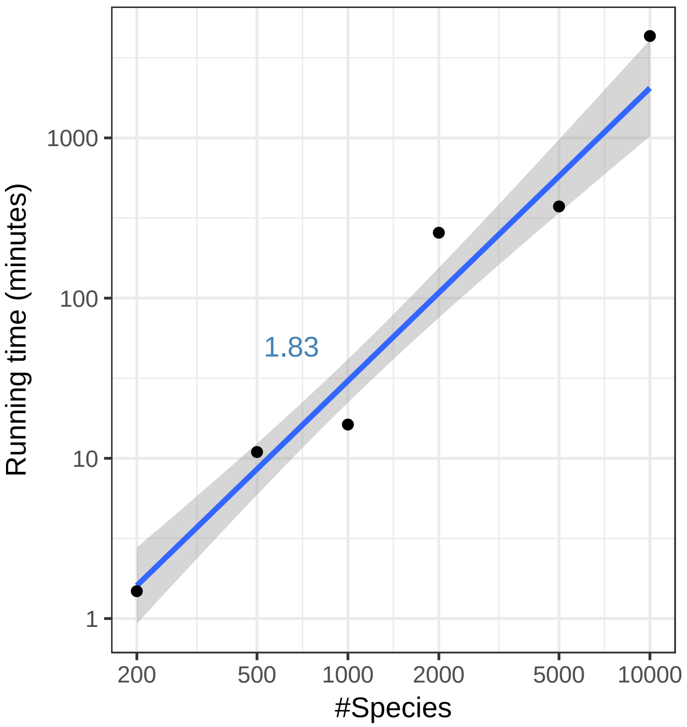

FIGURE S8. Empirical running time of ASTRAL-III with n. Average running time is shown for ASTRAL-III for datasets with varying n. Averages are over 20 replicates. One replicate of 2000 species dataset could not finish in 2 days and is removed from the analysis. Note that these datasets have factors other than n that change as well (e.g., the amount of ILS, etc.). Thus, these running times should be treated as ball-park estimates. Finally, we note that on the 10,000 dataset, we have only 2 replicates and not 20.
